# Characterization and analysis of multi-organ full-length transcriptomes in *Sphaeropteris brunoniana* and *Alsophila latebrosa* highlight secondary metabolism and chloroplast RNA editing pattern of tree ferns

**DOI:** 10.1186/s12870-024-04746-w

**Published:** 2024-01-26

**Authors:** Yang Peng, Zhen Wang, Minghui Li, Ting Wang, Yingjuan Su

**Affiliations:** 1 School of Life Sciences, Sun Yat-Sen University, Guangzhou, 510275 China; 2 Research Institute of Sun Yat-Sen University in Shenzhen, Shenzhen, 518057 China; 3 College of Life Sciences, South China Agricultural University, Guangzhou, 510642 China

**Keywords:** *Sphaeropteris brunoniana*, *Alsophila latebrosa*, Full-length transcriptome, Chloroplast genes, Environmental adaptation, RNA editing pattern

## Abstract

**Background:**

*Sphaeropteris brunoniana* and *Alsophila latebrosa* are both old relict and rare tree ferns, which have experienced the constant changes of climate and environment. However, little is known about their high-quality genetic information and related research on environmental adaptation mechanisms of them. In this study, combined with PacBio and Illumina platforms, transcriptomic analysis was conducted on the roots, rachis, and pinna of *S. brunoniana* and *A. latebrosa* to identify genes and pathways involved in environmental adaptation. Additionally, based on the transcriptomic data of tree ferns, chloroplast genes were mined to analyze their gene expression levels and RNA editing events.

**Results:**

In the study, we obtained 11,625, 14,391 and 10,099 unigenes of *S. brunoniana* root, rachis, and pinna, respectively. Similarly, a total of 13,028, 11,431 and 12,144 unigenes were obtained of *A. latebrosa* root, rachis, and pinna, respectively. According to the enrichment results of differentially expressed genes, a large number of differentially expressed genes were enriched in photosynthesis and secondary metabolic pathways of *S. brunoniana* and *A. latebrosa*. Based on gene annotation results and phenylpropanoid synthesis pathways, two lignin synthesis pathways (H-lignin and G-lignin) were characterized of *S. brunoniana*. Among secondary metabolic pathways of *A. latebrosa*, three types of WRKY transcription factors were identified. Additionally, based on transcriptome data obtained in this study, reported transcriptome data, and laboratory available transcriptome data, positive selection sites were identified from 18 chloroplast protein-coding genes of four tree ferns. Among them, RNA editing was found in positive selection sites of four tree ferns. RNA editing affected the protein secondary structure of the *rbcL* gene. Furthermore, the expression level of chloroplast genes indicated high expression of genes related to the chloroplast photosynthetic system in all four species.

**Conclusions:**

Overall, this work provides a comprehensive transcriptome resource of *S. brunoniana* and *A. latebrosa*, laying the foundation for future tree fern research.

**Supplementary Information:**

The online version contains supplementary material available at 10.1186/s12870-024-04746-w.

## Introduction

*Sphaeropteris brunoniana* Hook., an old tree-shaped fern with an arborescent trunk, belongs to the genus *Sphaeropteris* of Cyatheaceae [1]. *Alsophila latebrosa* Wall. Ex Hook., a palm-like tree fern, belongs to the genus *Alsophila* of Cyatheaceae. The two species are primarily distributed in tropical and subtropical lowlands and foothills [2–4]. The history of tree ferns can be traced back to the late Carboniferous to Triassic periods. Although many species became extinct in the late Permian, extant tree ferns originated from the Jurassic to Cretaceous periods [5–7]. Population statistics and bioclimatology researches indicate that members of the evolutionary branch of tree ferns have a longer generation time compared to non-tree lineages [8]. Among existing land plants, ferns are the closest lineage to seed plants, representing an ancient and highly diverse lineage [9]. Apart from existing seed plants, dendritic plants are mainly confined to tree ferns [10]. Tree ferns, which mostly have erect rhizome and have originated independently from different lineages [7]. The fossil record indicates that Cyatheaceae were the richest during the Jurassic period [1, 11]. Tree ferns are mainly composed of species of Cyatheaceae [1]. During the long evolutionary history, relict tree ferns have consistently adapted to changes in paleogeographic environments, forming many intricate mechanisms to respond to various environmental stresses, which provide huge value for further adaptive evolution.

Compared with the majority of seed plants, ferns have larger genomes, with an average size of 12 Gb [12], some of which can be up to 148 Gb [13]. Therefore, the whole genome sequencing of fern species is extremely challenging. Given that the amount of transcriptome data is smaller than the genome and contains rich genetic information, it is a highly feasible method to study ferns by transcriptome sequencing. However, transcriptome sequencing of single tissue or organ cannot provide comprehensive gene information [14], sequencing of different organs and tissues of the same species is conducive to enriching the diversity of transcripts in the organism and discovering genes specifically expressed in different organs and tissues [15]. Therefore, an increasing number of transcriptome studies are focused on organ and tissue differences analysis. Among the fern transcriptome researches, only a few full-length transcriptomes of tree fern species have been reported, including *Ceratopteris richardii* [16], *A. spinulosa* [7], *Drynaria roosii* [17] and *Pteris vittata* [18]. The full-length transcriptome data obtained for the first time in this study will greatly contribute to enriching the gene information and transcriptome resources of *S. brunoniana* and *A. latebrosa*.

Perennials often confront adverse environmental conditions during their growth and development [19–21]. In response to these challenges, early land plants have evolved a series of specialized metabolic pathways, known as secondary metabolism [22]. Phenylpropanoid metabolism, in particular, plays a critical role in metabolic pathways for ferns to adapt to the environment [23]. Lignin synthesis is closely associated with the erect rhizome of tree ferns. Lignin, as one of the most prominent products of phenylpropanoid synthesis pathway, provides an upright rigid structure for vascular plants and strengthens the cell wall of water-conducting molecules, enabling them to withstand the negative pressure generated during transpiration, thus further adapting to the environment [22]. Therefore, lignin biosynthesis has been considered as one of the vital factors for the development of terrestrial plants in terrestrial ecosystems. Ferns, as one of the earliest vascular plants, were closely related to lignin synthesis for their early abundant species diversity. In addition, plant hormones are widely present in plants and produced in secondary metabolism [24]. As signal molecules, they function through various pathways and give plants plasticity to adapt to the changing growth and development environment [25]. Transcription factors (TFs) are involved in the regulation of plant secondary metabolite synthesis. As one of the largest transcriptional regulator families in plants, WRKY transcription factors involve in plant growth, immunity, and regulatory signaling networks [26]. According to Bakshi et al. [27], the WRKY gene family was generated through gene duplication during evolution, through the study of the phylogenetic relationship of the WRKY domain. They perform pivotal functions in regulating of plant physiological development and orchestrating stress responses. Therefore, the analysis of hormone levels and transcription factor families in ferns yields valuable molecular resources for subsequent study of hormone regulation mechanisms.

With the continuous development of sequencing technology, the understanding of the structure of nuclear transcripts has continued to deepen. However, another major component of transcriptome data, organelle gene sequences, has been almost ignored. In fact, organelle transcripts usually account for a large proportion of eukaryotic RNA-Seq results [28]. Transcriptome data have obvious advantages in chloroplast gene mining. Firstly, the gene expression level in organelles is generally higher than that in nuclear genes, eukaryotic RNA-Seq data contain a large number of organelle genes [29]. Secondly, as organelle genomes are generally transcribed into polycistronic RNAs, it is reasonable and possible to obtain abundant and high-quality organelle genes from transcriptome data [30]. However, mining the correct chloroplast genes from RNA-Seq data is challenging. Although Illumina sequencing technology has high single-base accuracy, the length of the DNA fragments it produces is usually around 50–400 bp [31], which is smaller than the length of most chloroplast genes. With the emergence of third-generation sequencing technologies, such as SMRT sequencing by PacBio, typically producing fragments larger than 10 kb [31], a single read can cover the entire chloroplast genome, or at least a large portion of it. Ultra-long fragments facilitate de novo inference of chloroplast structure, especially for chloroplast genomes with atypical structures [32], which indicates that the mining of chloroplast genes using long reads is efficient and feasible.

Chloroplast genes, especially those involved in photosystems, play a crucial role in environmental adaptation. Previous research has indicated that RNA editing events in chloroplasts modify the RNA sequence through base modifications to enhance the diversity of gene products [33, 34]. This mechanism regulates the functionality of chloroplast genes, which facilitates plant adaptation to the environment [35]. RNA editing often occurs in chloroplasts, and its editing process exhibits a diverse molecular diversity, some of which appear to be evolutionarily recently acquired and independently produced, and its type is generally a highly specific transformation from cytidine to uridine [36]. At present, the discovery of RNA editing in chloroplasts provides researchers with a large number of molecular and evolutionary puzzles, many of which remain unsolved. There is no unified conclusion on the origin and evolution of RNA editing, and there are different perspectives. One theory is based on random genetic drift, which suggests that the emergence and fixation of mutations at editable sites are primarily influenced by random genetic drift. It is also proposed that natural selection may play a role in maintaining RNA editing activity [37]. Adaptive editing theory [38] suggests that the purpose of editing is to correct or repair gene sequence defects, acting as a repair mechanism. According to the enzyme mutation view, RNA editing is initiated by mutations in enzymes capable of deamination or transamination [39, 40]. With this ability established, thymidine nucleotides in the genome can be replaced by cytidine, correcting the information content in RNA [41]. Furthermore, an argument proposes that RNA-edited sequences, which are generated through C to U editing for initiating codons or U to C editing for removing termination codons in various plant organelles and mitochondria, have advantages in translation compared to sequences encoded by the genome. This suggests that these specific RNA editing events are not selectively neutral and supports the idea that RNA editing functions as a control mechanism for gene expression in fern organelles [12].

Here, we combined the PacBio Iso-Seq and Illumina RNA-Seq technologies to reliably perform comprehensive transcriptome analyses and characterize the gene expression profiles in three organs of *S. brunoniana* and *A. latebrosa*. The aims of our study include: (i) generating reference transcriptome sequences for *S. brunoniana* and *A. latebrosa* from three organ by using the PacBio Iso-Seq technique; (ii) exploring gene expression patterns and differentially expressed genes (DEGs) among the three organs; (iii) identifying candidate genes and secondary metabolic pathways for adaptation to biotic and abiotic factors; and (iv) calculating the expression levels of chloroplast genes, and analyzing the relationship between RNA editing events in chloroplast genes and adaptive evolution. This work was the first comprehensive report on the full-length transcriptome of multiple organs of *S. brunoniana* and *A. latebrosa*, which provide a valuable molecular-level reference for future studies on the functional genomics, adaptive evolution, phylogeny, and conservation of *S. brunoniana*, *A. latebrosa* and other tree ferns.

## Results

### The full-length sequences of PacBio Iso-Seq

Through the PacBio Sequel sequencing of *S. brunoniana*, polymerase reads of 16.28 Gb, 25.11 Gb, and 18.61 Gb were obtained in root, rachis, and pinna, respectively. After filtering low-quality sequences, the subreads obtained were 15.36 Gb (root), 23.82 Gb (rachis), and 17.62 Gb (pinna), with average lengths were 1,239 bp, 1,430 bp, and 1,367 bp, respectively (Table S1). In order to further improve the quality of the transcript sequence, data correction and 95% sequence similarity redundancy analysis were performed, in the transcriptome of *S. brunoniana*. Totally, 11,625 unigenes were obtained in the root, with a major concentration of 1,000–2,000 bp (6,485). A total of 14,391 unigenes were obtained from the rachis, of which 62.7% (9,027) unigenes were 1,000–2,000 bp in length. A total of 10,099 unigenes were identified in the pinna (Table 1). In addition, the N50 value of unigenes were 1,594 bp (root), 1,714 bp (rachis) and 1,591 bp (pinna), respectively.

**Table 1:** Unigenes statistics of *S. brunoniana* and *A. latebrosa*

Species	Organs	< 500 bp	500—1,000 bp	1—2 kb	2 – 3 kb	> 3 kb	Total
*S. brunoniana*	Root	129	3,166	6,485	1,651	194	11,625
	Rachis	31	2,259	9,027	2,731	343	14,391
	Pinna	9	1,862	6,676	1,441	111	10,099
*A. latebrosa*	Root	60	3,411	7,549	1,829	179	13,028
	Rachis	9	1,028	7,358	2,625	411	11,431
	Pinna	62	2,448	7,047	2,284	303	12,144

The SMRT sequencing of *A. latebrosa* yielded 18.90 Gb (root), 18.57 Gb (rachis), and 21.52 Gb (pinna) of polymerase reads, respectively. After filtering, 18.02 Gb, 17.58 Gb, and 20.24 Gb of subreads were obtained from the root, rachis, and pinna, respectively, and the N50 was 1,663bp, 1536bp, and 1,318bp, respectively (Table S2). Following correction and redundancy analysis, 13,028 unigenes, 11,431 unigenes and 12,144 unigenes were obtained from the root, rachis and pinna, respectively (Table 1). The N50 of unigenes from the root, rachis and pinna were 1,818 bp, 1,720 bp and 1,581 bp, respectively.

### De novo assembly of Illumina RNA-Seq data

The Illumina RNA-seq generated 42,150,438 (root), 47,496,118 (rachis), and 60,482,180 (pinna) raw reads. After trimming and filtering, the root, rachis, and pinna samples yielded 6.19 Gb, 6.97 Gb, and 8.78 Gb of clean reads, respectively. The GC content in all samples ranged between 49 and 51% (Table S3). Consequently, a total of 41,816 unigenes, 27,159 unigenes, and 36,429 unigenes were obtained from the root, rachis, and pinna, respectively, based on these clean reads, with N50 sizes of 1,631 bp, 1,962 bp and 1,628 bp, respectively (Table S4).

For Illumina sequencing of *A. latebrosa*, 42,150,438 (root), 47,496,118 (rachis), and 60,482,180 (pinna) raw reads were generated. After quality control, 8.03 Gb, 6.47 Gb, and 6.89 Gb clean reads were obtained for the root, rachis, and pinna, respectively (Table S5). Clean reads were independently assembled to 33,487 unigenes, 27,031 unigenes and 28,770 unigenes from root, rachis and pinna, with N50 values of 1,916 bp, 1,910 bp and 1,854 bp, respectively (Table S6). Overall, the sequencing results indicated that the Illumina data obtained in this study was of high quality.

### Gene function annotation

To derive the most information and obtain a comprehensive annotation of *S. brunoniana* and *A. latebrosa* transcriptome, we performed a similarity search using these sequences by searching against seven databases, including the NCBI Nr database (https://www.ncbi.nlm.nih.gov/protein/), KOG database (ftp://ftp.ncbi.nih.gov/pub/COG/KOG/), Swiss-Prot database (https://www.uniprot.org/uniprot/), KEGG database (http://www.genome.jp/kegg/), NCBI nucleotide sequences (Nt) database, GO (http://www.geneontology.org/) database and Pfam database (https://pfam.xfam.org).

Among the three organs of *S. brunoniana*, the majority of unigenes in the transcriptome were annotated in Nr, SwissProt and KEGG database. For the root transcriptome, 10,454 genes were annotated by at least one database, and 2,527 genes were annotated by all seven databases. In the rachis transcriptome, 13,592 genes and 3,466 genes were annotated by at least one database and seven databases, respectively. Of the pinna transcriptome, 9,559 genes were annotated by at least one database, and 2,355 genes were annotated by all seven databases. Overall, genes were successfully annotated in 90% (root), 94% (rachis) and 95% (pinna) of the three organ transcriptomes, as shown in Table 2.

**Table 2:** Gene functional annotations of *S. brunoniana* and *A. latebrosa*

Species	*S. brunoniana*			*A. latebrosa*		
Database	Root	Rachis	Pinna	Root	Rachis	Pinna
Nr	9,951	13,314	9,314	10,591	11,043	11,716
SwissProt	8,700	11,534	8,041	9,163	9,541	9,958
KEGG	9,724	13,173	9,176	10,413	10,902	11,533
KOG	6,677	8,753	5,920	6,960	7,176	7,205
GO	7,617	9,940	6,912	7,937	8,237	8,572
Nt	3,726	5,154	3,547	4,160	4,278	4,289
Pfam	7,617	9,940	6,912	7,937	8,237	8,572
At least one database	10,454	13,592	9,559	10,808	11,321	12,066
All databases	2,527	3,466	2,355	2,802	2,899	2,777

**Fig. 1:**
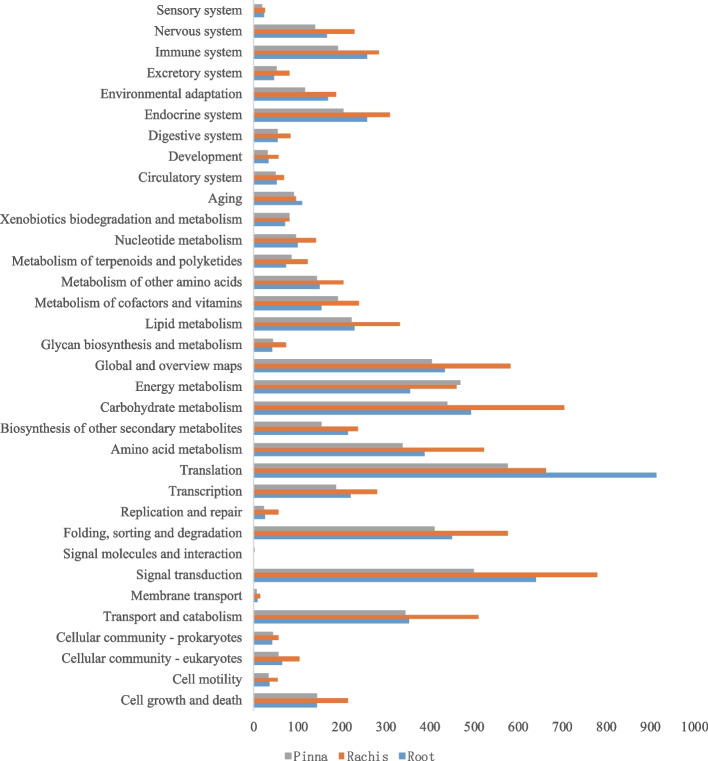
KEGG enrichment results of unigenes in *S. brunoniana*

Due to the limited availability of fern genome resources, only a few unigenes have been annotated for fern species from the Nr database. In this study, the majority of unigenes were annotated to bryophytes and gymnosperms (Fig. S1). According to the results of the KEGG enrichment analysis, the unigenes from the transcriptome of the root, rachis and pinna were mapped to 282, 353 and 345 metabolic pathways, respectively (Table S7, Fig. 1). For root, rachis, and pinna of *A. latebrosa*, most of the transcriptome genes in the three organs were annotated. 10,808, 11,321, and 12,066 unigenes were annotated to at least one database, respectively. These results support the comprehensive annotation of our transcriptome and suggest that the majority of unigenes have functional roles (Table 2).

### Analysis of TF, lncRNA and SSR

**Fig. 2:**
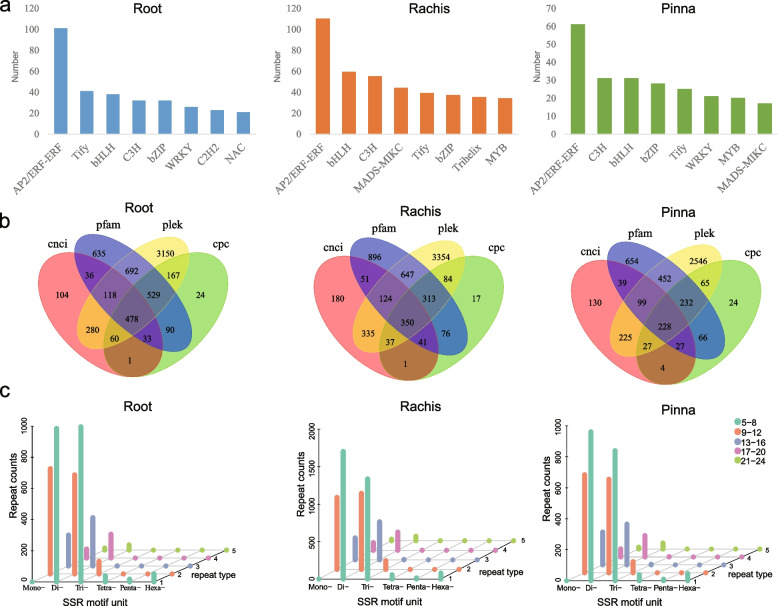
Structural prediction of three-organ full-length transcriptomes of *S. brunoniana.* **a** Transcription factor family distribution (top eight). **b** The quantity of lncRNAs in three organs. **c** Distribution of SSR motifs. The X axis represents the SSR motif units, i.e., the number of repeating bases. The Y axis represents the number of repetitions of the bases, where the specific repetition count corresponds to the colors mentioned in the legend. The Z axis represents the number of SSRs

Based on prediction and statistics from transcriptome data of *S. brunoniana*, the AP2/ERF-ERF family was found to be the most abundant TF family in the root, rachis and pinna transcriptome (Fig. 2a). A total of 6,397 long non-coding RNAs (lncRNAs) were identified from the root transcriptome of *S. brunoniana* by PLEK, CNCI and CPC software. A total of 6,506 lncRNAs were identified from the rachis transcriptome, while 4,818 lncRNAs were identified from the pinna transcriptome (Fig. 2b). We identified 4,294, 6,520 and 3,926 single sequence repeats (SSRs) in the root, rachis and pinna transcriptome of *S. brunoniana*, respectively. Among the SSRs identified in the three organs, dinucleotide repeats were the most common SSRs, with 2,117 in roots, 3,531 in rachis, and 2,005 in pinna. For root, the most abundant type of SSRs was trinucleotide with a repeat number of 5–8, totaling 996. For rachis and pinna, the most abundant type of SSRs was dinucleotide with a repeat number of 5–8, totaling 1704 and 961 respectively. (Fig. 2c).

A total of 4,880 lncRNAs were identified in the root transcriptome of *A. latebrosa*. The rachis transcriptome contained 5,697 lncRNAs, while the pinna transcriptome contained 6,772 lncRNAs (Fig. S2a). Based on the results of TF identification, the five largest transcription factor families in *A. latebrosa* transcriptome were AP2/ ERF-ERf family, bHLH family, C3H family, Tify family and bZIP family (Fig. S2b). In addition, from the transcriptomes of the three organs of *A. latebrosa*, 4,918 (root), 5,350 (rachis), and 5,292 (pinna) SSRs were identified. Among them, the most abundant type of SSRs was the dinucleotide repeat type, accounting for approximately 57%. Regarding the mononucleotide repetition types, the most frequently repeated type occurred 9–12 times. In the range from dinucleotide repeat type to hexanucleotide repeat type, the most repeated types were 5–8 times (Figure S4).

### Gene expression level and enrichment analysis of differentially expressed genes of different organs

To investigate the expression patterns of unigenes in *S. brunoniana* and *A. latebrosa*, the Illumina clean reads were aligned to the SMRT non-redundant transcripts to determine expression level using FPKM (expected number of fragments per kilobase of transcript sequence per millions base pairs sequenced). The mapping rates of each organ in *S. brunoniana* were 70.92% (root), 78.12% (rachis), and 73.93% (pinna), respectively. In *A. latebrosa*, the mapping rates were 76.82% (root), 80.57% (rachis) and 79.19% (pinna), respectively (Table 3).

**Table 3:** Alignment of clean reads and consensus sequences

Species	Samples	Total reads	Total mapped	Mapping rates
*S. brunoniana*	Root	41,283,200	29,278,466	70.92%
	Rachis	46,476,174	36,307,450	78.12%
	Pinna	31,525,522	23,306,874	73.93%
*A. latebrosa*	Root	53,522,458	41,115,214	76.82%
	Rachis	43,137,240	34,755,608	80.57%
	Pinna	45,924,740	36,369,062	79.19%

**Fig. 3:**
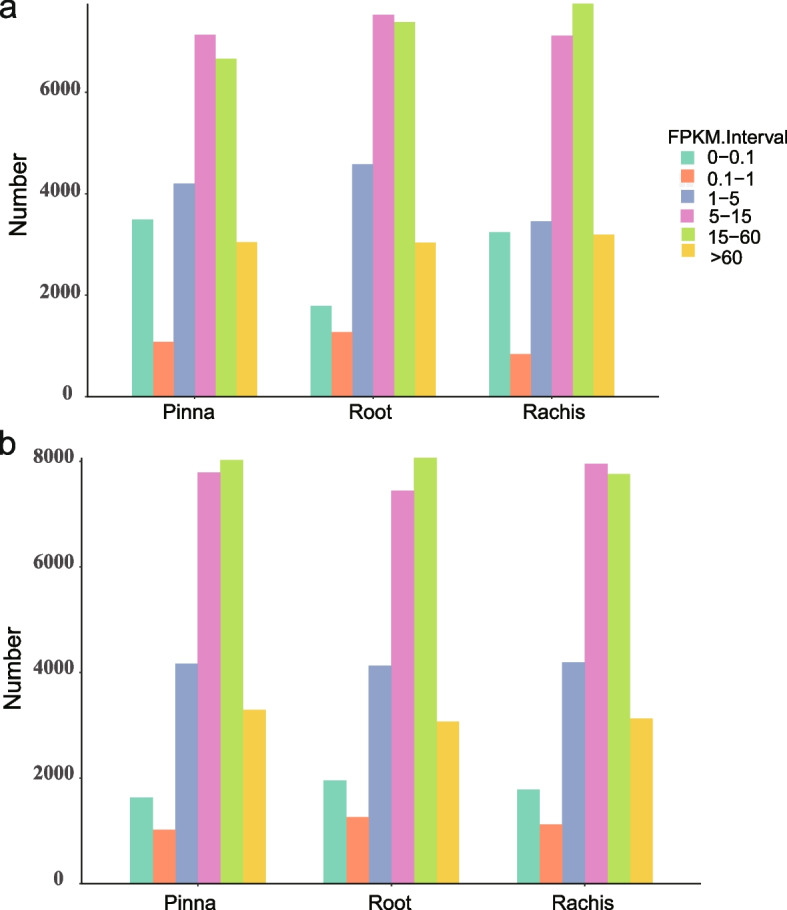
Gene number statistics with different FPKM values (**a**) *S. brunoniana*, (**b**)* A. latebrosa*

In the three organs of *S. brunoniana*, FPKM interval analysis showed that the FPKM values between 15 and 60 accounts for 28.37% of all unigenes in three organs, followed by FPKM values between 5 and 15, accounting for 28.36% of all unigenes (Table S8, Fig. 3). In the three organs of *A. latebrosa*, FPKM value was mainly concentrated in 5–60 (Table S9, Fig. 3).

With FPKM > 0.3 as the standard, differentially expressed genes among samples were analyzed. In the transcriptome of *S. brunoniana*, the highest number of differentially expressed genes (8,700) was observed between the pinna and root. The differences between pinna and rachis, as well as between root and rachis, were relatively small, with 5,896 and 5,195 DEGs, respectively. In the transcriptome of *A. latebrosa*, the number of DEGs between pinna and root was the highest (8,076), and the number of DEGs between root and rachis was the lowest (3,308) (Table 4). Thus, the difference between the root and the rachis is relatively large, whereas the differences between root and rachis are relatively small.

**Table 4:** Number of differentially expressed genes between pairs of samples

Species	Samples	DEGs	Up-expression genes	Down-expression genes
*S. brunoniana*	Pinna vs Root	8,076	4,210	3,866
	Root vs Rachis	3,308	1,687	1,621
	Pinna vs Rachis	5,877	3,248	2,629
*A. latebrosa*	Pinna vs Root	8,700	3,893	4,807
	Root vs Rachis	5,195	2,738	2,457
	Pinna vs Rachis	5,896	2,651	3,245

To comprehensively analyze the functions of DEGs, GO and KEGG enrichment were conducted on differentially expressed genes. In *S. brunoniana*, GO enrichment of DEGs between pinna and root enriched 53 pathways, among which metabolic process (GO:0008152) and catalytic activity (GO:0003824) contained the largest number of unigenes, 3,318 and 2,952, respectively (Table S10). The DEGs enrichment between root and rachis resulted in a total of 41 differentially expressed GO pathways (Table S11). Additionally, the GO enrichment of DEGs between pinna and rachis revealed 54 pathways (Table S12). For *A. latebrosa*, there were 55 GO terms enriched in the DEGs between pinna and root (Table S13). Furthermore, the DEGs between root and rachis enriched a relatively small number of GO terms, only 39 (Table S14). Similar to the enrichment results of DEGs between pinna and root, DEGs between pinna and rachis were also mainly enriched in metabolic process and catalytic activity, with over 2,000 genes enriched (Table S15).

The results of KEGG enrichment on DEGs showed that the up-regulated genes in the transcriptome of *S. brunoniana* and *A. latebrosa* were mainly belonged to photosynthetic pathways (Fig. S3-Fig. S8). In addition, a large number of up-regulated genes in root and rachis were found to be associated with secondary metabolism-related pathways.

### Lignin synthesis pathway in *S. brunoniana*

**Fig. 4:**
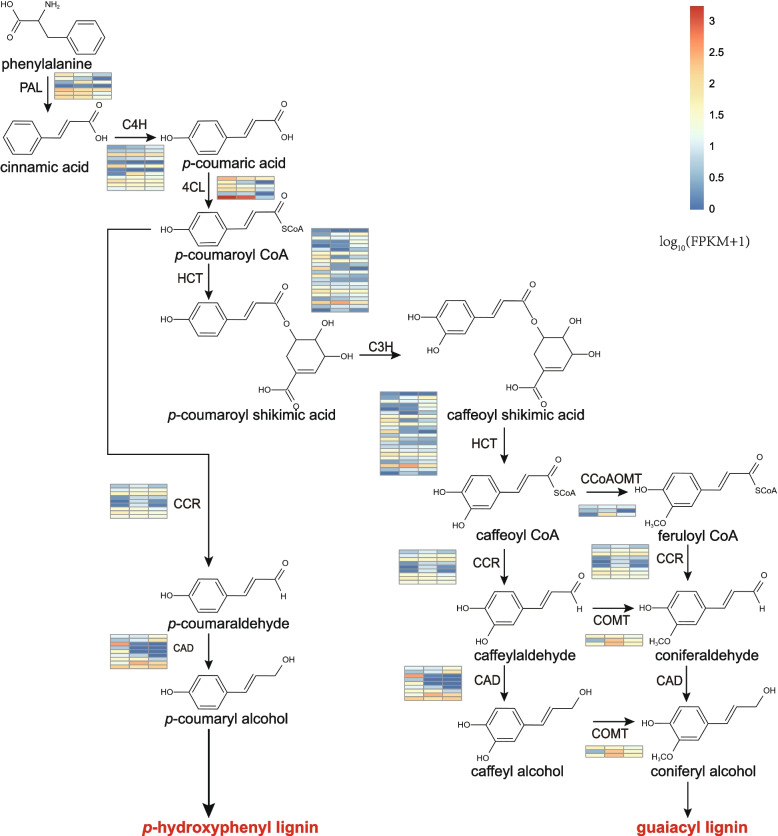
Pathway of lignin synthesis of *S.brunoniana* (The colors in the square are the gene expression level of the root, rachis and pinna)

It was found that there were two lignin synthesis pathways in *S. brunoniana*: P-hydroxyphenyl lignin (H-lignin) and Guaiacyl lignin (G- lignin) synthesis pathway (Fig. 4). The absence of Syringyl lignin (S-lignin) synthesis pathway was attributed to the absence of ferulate-5-hydroxylase (F5H) in ferns [42]. In the lignin synthesis pathway, the expression levels of *PAL* and *4CL* in roots and rachis of *S. brunoniana* were higher. *CCR*, *CCoAOMT* and *COMT* showed high expression in rachis. The expression level of *CAD* was higher in roots.

**Fig. 5:**
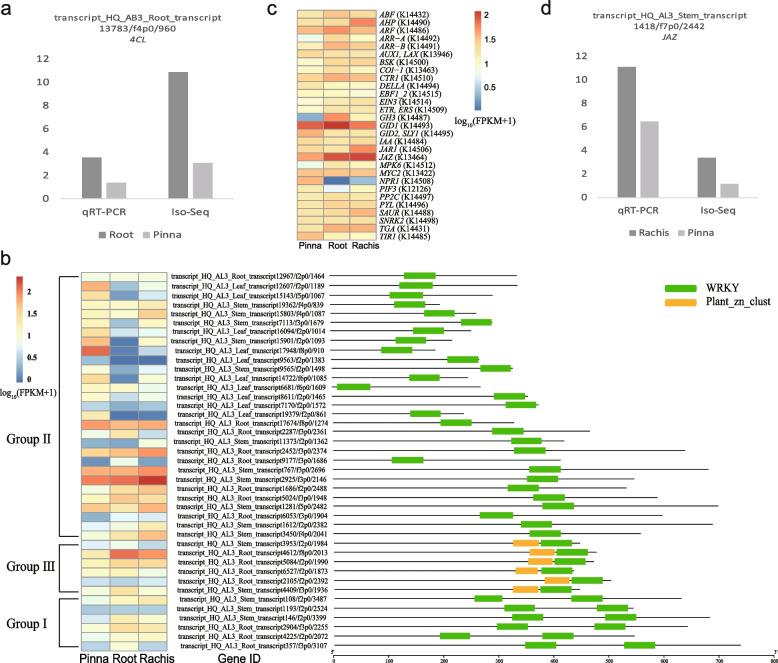
Characterization of genes involved in the secondary metabolic pathways of *S.brunoniana* and *A. latebrosa*. **a** *4CL* unigene of *S.brunoniana* validation by qRT-PCR. **b** Protein motifs and expression levels of WRKY transcription factor of *A. latebrosa*. **c** Gene expression levels of hormone signal transduction pathway of *A. latebrosa*. **d** *JAZ* unigene of *A. latebrosa* validation by qRT-PCR

To ensure the accuracy of the gene expression analysis, we performed qRT-PCR to validate the *4CL* gene (transcript_HQ_AB3_Root_transcript13783/f4p0/960). By using the rachis as control sample, the expression pattern of the *4CL* gene in Iso-Seq analysis was correlated with the qRT-PCR assay (Fig. 5a). Both analyses revealed significantly higher expression levels in the root compared to the pinna. The results of qRT-PCR confirm the quantitative analysis of gene expression. Detailed information regarding the qRT-PCR experiment and the primer pairs can be found in Supplementary Table S16.

### WRKY transcription factors and signal transduction pathway of *A. latebrosa*

Based on the predicted structure of transcription factors, 85 WRKY transcription factor family members were identified from the transcriptomes of three organs of *A. latebrosa*, and 41 gene sequences were obtained by deleting unexpressed gene sequences and incomplete protein motifs, and eliminating redundancy with 95% similarity (Table S17). According to the classification method of Eulgem et al. [26, 43] and prediction results of HMMER motif, three types of WRKY transcription factors were identified. Among them, the type containing the largest number of transcription factor members was Group II, which contained 29 WRKY transcription factors, and the members of this type only contained one WRKY domain. The number of family members of Group III type and Group I type was six. The structure of Group III contained a WRKY domain and a zinc finger domain. The structural characteristics of Group I type included two WRKY domains (Fig. 5b). By analyzing the expression levels of identified WRKY transcription factor family members, the expression level of WRKY gene in roots was generally lower than that in pinna and rachis in Group II. In Group III gene, the expression level of members in root and rachis was slightly higher than that in pinna. Among the genes of Group I, the expression levels of WRKY transcription factor family members in various organs were slightly different, and the gene expression levels in root and rachis were slightly higher than that in pinna.

**Fig. 6:**
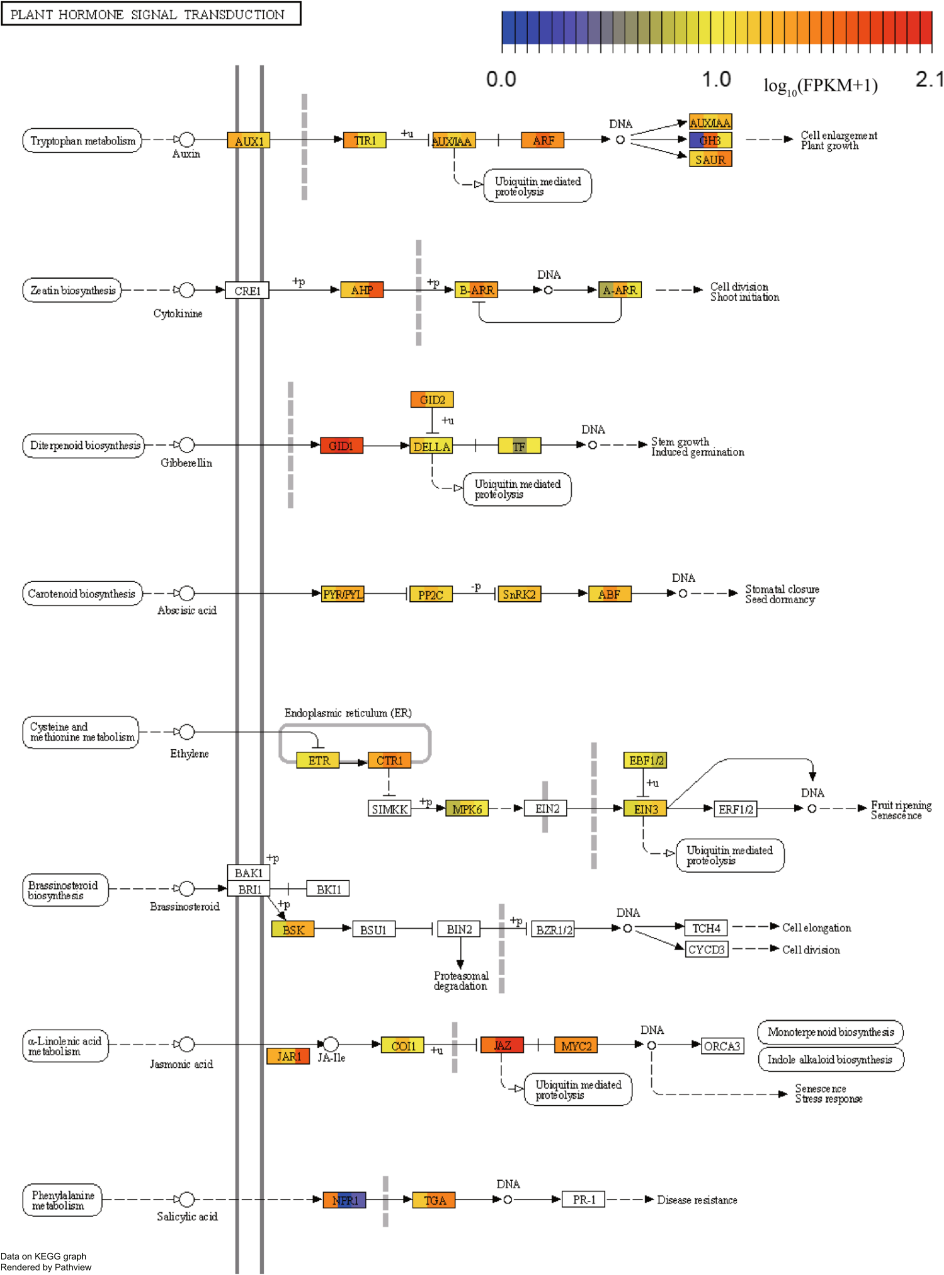
Plant hormone signal transduction pathways of *A. latebrosa* (the colors in the boxes represent the levels of gene expression of the pinna, root and rachis). The pathways were derived from the KEGG map and rendered by Pathview

Based on KEGG annotation, a total of 463 unigenes were assigned to the plant hormone signal transduction pathway (KO 04075), encoding organics in this pathway (Table S18). The expression levels of genes involved in hormone signal transduction were calculated. On the whole, the expression levels of genes involved in hormone signal transduction pathway were higher. *GID1* gene encoding gibberellin receptor and *JAZ* gene encoding jasmonic acid ZIM domain protein were highly expressed in *A. latebrosa*, especially in root and rachis. However, the expression of *NPR1* gene encoding regulatory protein was relatively low in root and rachis (Fig. 5c). The hormone signal transduction pathways of *A. latebrosa* mainly include Gibberellin, Jasmonic acid, Abscisic acid and Salicylic acid (Fig. 6).

To verify the accuracy of the analysis of gene expression, a JAZ gene (transcript_HQ_AL3_Stem_transcript1418/f7p0/2442) was confirmed through qRT-PCR. Using the root as the control sample, the expression pattern of the JAZ gene in the Iso-Seq analysis was compared to the qRT-PCR assay (Fig. 5d). Both indicated significantly higher expression levels in the rachis compared to the pinna. The results of the qRT-PCR validate the quantitative analysis of gene expression. The experimental results and primer pairs used in the qRT-PCR can be found in Supplementary Table S14.

### Phylogenetic tree

**Fig. 7:**
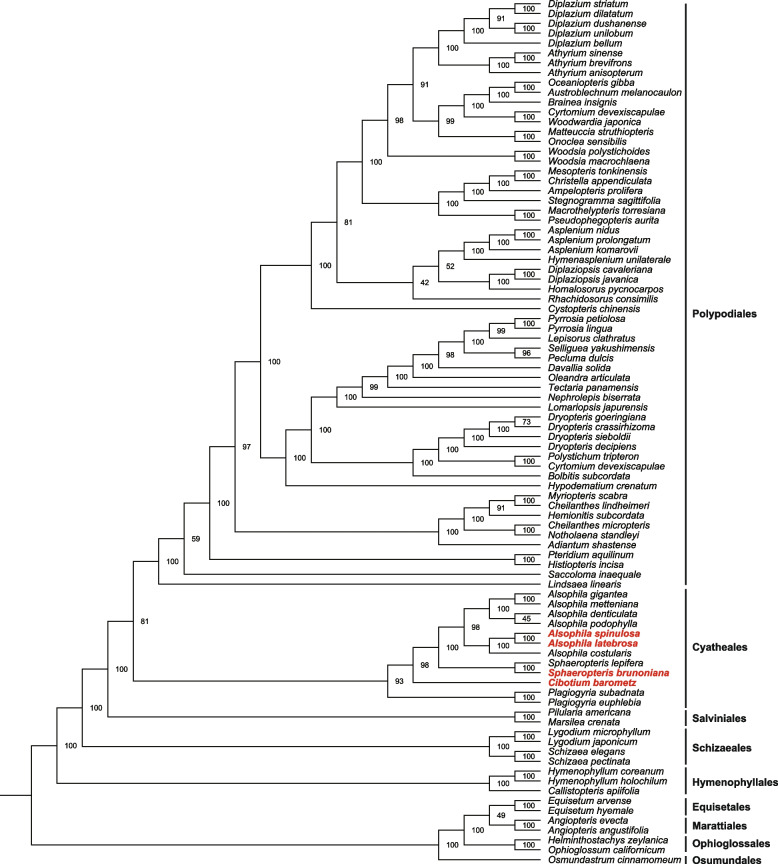
Phylogenetic tree based on 18 protein-coding genes

Phylogenetic tree of 88 fern species was constructed using tandem datasets of 18 protein-coding genes based on the GTRGAMMA model (Fig. 7). Referring to the classification of PPG I (The Pterido-Phyte Phylogeny Group) [1] and the classification of existing ferns published by Smith et al. [44], the phylogenetic tree constructed in this study was basically consistent with the accepted fern phylogenetic tree. *Alsophila. latebrosa* and *A. spinulosa* clustered in the same branch and belonged to the genus *Alsophila*. The branches of *S. brunoniana*, *A. latebrosa*, *A. spinulosa*, and *Cibotium barometz* were grouped together and belong to the order Cyatheales. In addition, the main function of this phylogenetic tree was to provide tree files for subsequent adaptive evolutionary analysis.

### Adaptive evolution analysis

The adaptive evolution of 18 chloroplast genes was analyzed using the site model of PAML codeml program. Based on M1a and M2a models, a total of 8 positive selection sites were identified, which distributed in *atpH, psbE, psbL, rbcL* and *rpl32* genes. However, as the* P* value of *rbcL* gene based on likelihood ratio test was greater than 0.01, this positive selection site was rejected, so a total of 7 positive selection sites were obtained (Table 5). Based on M7 and M8 models, a total of 18 positive selection sites were identified, which were distributed in *atpH, psaA, psaB, psbB, psbE, psbL, psbK, rbcL*, *rps14* and *rpl32* genes. As the *P* value of M7-M8 model likelihood ratio test of *psbK* gene and *rps14* gene was greater than 0.01, positive selection sites identified in these two genes were rejected and a total of 16 positive selection loci were obtained (Table 6).

**Table 5:** Likelihood ratio test and positive selection sites based on M1a model and M2a model

Genes	D.f	2Δℓ	*P*-value	Positive selection sites
*atpH*	2	14.704	6.412 × 10^–4^	4L* 75L**
*psaA*	2	0.000	1.000	None
*psaB*	2	0.000	1.000	None
*psbB*	2	0.000	1.000	None
*psbC*	2	0.000	1.000	None
*psbD*	2	0.000	1.000	None
*psbE*	2	10.880	4.339 × 10^–3^	30L**
*psbF*	2	0.327	0.849	None
*psbH*	2	0.000	1.000	None
*psbI*	2	0.203	0.904	None
*psbK*	2	0.000	1.000	None
*psbL*	2	11.921	2.579 × 10^–3^	13S* 31L**
*psbT*	2	0.142	0.931	None
*rbcL*	2	0.001	1.000	251L*
*rpl21*	2	4.803	0.091	None
*rpl32*	2	41.609	9.222 × 10^–3^	63P* 64S**
*rps14*	2	3.017	0.221	None
*ycf12*	2	0.000	1.000	None

Annotations: ** *P* > 99%; * *P* > 95%

**Table 6:** Likelihood ratio test and positive selection sites based on M7 model and M8 model

Genes	D.f	2Δℓ	*P*-value	Positive selection sites
*atpH*	2	30.473	2.410 × 10^–5^	4L** 55L* 74L** 75L**
*psaA*	2	23.493	7.916 × 10^–6^	209G*
*psaB*	2	15.817	3.677 × 10^–4^	255L*
*psbB*	2	31.927	1.167 × 10^–7^	238L*
*psbC*	2	31.401	1.518 × 10^–7^	None
*psbD*	2	1.977	0.337	None
*psbE*	2	38.746	3.858 × 10^–9^	30L**
*psbF*	2	5.620	0.060	None
*psbH*	2	0.001	0.999	None
*psbI*	2	7.377	0.025	None
*psbK*	2	8.233	0.016	10 M*
*psbL*	2	32.383	9.290 × 10^–8^	13S** 24L* 31L**
*psbT*	2	2.364	0.307	None
*rbcL*	2	31.228	1.655 × 10^–7^	116 M* 251L* 375L**
*rpl21*	2	4.590	0.101	None
*rpl32*	2	44.376	2.311 × 10^–10^	63P** 64S**
*rps14*	2	6.445	0.040	25S*
*ycf12*	2	2.987	0.225	None

Annotations: ** *P* > 99%; * *P* > 95%

In a likelihood ratio test for near-neutral model M1a and selective model M2a, the double logarithmic likelihood value of the *atpH* gene 2Δℓ was 14.704, with a *P*-value of 6.412 × 10^–4^, two positive selection sites were identified, leucine at position 4 and leucine at position 75, of which leucine at position 75 has a posteriori probability of more than 99%. In the *psbE* gene, a positive selection site (30L) was identified with a posteriori probability of more than 99%. In *psbL* gene, the *p*-value of likelihood ratio test was 2.579 × 10^–3^, and two positive selection sites (13S and 31L) were screened out, of which 31L had a posteriori probability of over 99%. In the case of the *rpl32* gene, a total of two positive selection sites (63P and 64S) were identified, with serine at position 64 having a posteriori probability of more than 99%.

In the likelihood ratio test of M7 model and M8 model, a total of 4 positive selection sites (4L, 55L, 74L and 75L) were identified in *atpH* gene, and the *P* value of likelihood ratio test was 2.410 × 10^–5^. The leucine posterior probabilities of the 4th, 74th and 75th sites were all greater than 99%. In *psbL* gene, three positive selection sites (13S, 24L, and 31L) were identified, of which 13S and 31L had a posteriori probability of more than 99%. In *rbcL* gene, the *p*-value of likelihood ratio test was 1.655 × 10^–7^, and three positive selection sites (116M, 251L and 375L) were screened out, of which 375L had a posteriori probability of more than 99%. In *rpl32* gene, two positive selection sites were identified, proline at 63 and serine at 64, with a posteriori probability of more than 99%. A positive selection site of 209G, 255L, 238L and 30L was screened for *psaA*, *psaB*, *psbB* and *psbE*, respectively. Among them, only leucine, the 30th position of *psbE* gene, had a posterior probability greater than 99%, and the rest were all greater than 95%.

### RNA editing sites

Among the 16 positive selection sites, RNA editing was identified in 4, 5, 6 and 5 positive selection sites of *S. brunoniana*, *A. latebrosa*, *A. spinulosa*, and *C. barometz*, respectively (Table 7). RNA editing occurred in 75L of *atpH*, 238L of *psbB*, 30L of *psbE* and 31L of *psbL* in all four species. In addition, C-U editing also occurred in 255L of *psaB* of *A. latebros*, 255L of *psaB* gene of *C. barometz*, 255L of *psaB* and 375F of *psaB* of *A. spinulosa*. In *atpH, psaB* and *psbB*, the codon before RNA editing was TCA, encoding Serine (S). After editing by C-U, the codon was TTA encoding Leucine (L). In *psbE*, the CCG codon encoding Proline (P) was edited by RNA, and the codon was CTG encoding Leucine. In *rbcL*, the site encoding leucine (CTT) was changed to the site encoding Phenylalanine (F) (TTT) by RNA editing. In *psbL*, the codon before RNA editing was CCA, encoding Proline. After editing by C-U, the codon was CTA encoding Leucine. In addition, all the RNA editing types identified in this study were C-U editing, which mainly occurred at the second codon position.

**Table 7:** RNA editing at positive selection sites

Species	Genes	Positive selection sites	RNA editing	Type
*S. brunoniana*	*atpH*	75	TCA (S) → TTA (L)	C-U editing
	*psbB*	238	TCA (S) → TTA (L)	C-U editing
	*psbE*	30	CCG (P) → CTG (L)	C-U editing
	*psbL*	31	CCA (P) → CTA (L)	C-U editing
*A. latebrosa*	*atpH*	75	TCA (S) → TTA (L)	C-U editing
	*psaB*	255	TCA (S) → TTA (L)	C-U editing
	*psbB*	238	TCA (S) → TTA (L)	C-U editing
	*psbE*	30	CCG (P) → CTG (L)	C-U editing
	*psbL*	31	CCA (P) → CTA (L)	C-U editing
*A. spinulosa*	*atpH*	75	TCA (S) → TTA (L)	C-U editing
	*psaB*	255	TCA (S) → TTA (L)	C-U editing
	*psbB*	238	TCA (S) → TTA (L)	C-U editing
	*psbE*	30	CCG (P) → CTG (L)	C-U editing
	*psbL*	31	CCA (P) → CTA (L)	C-U editing
	*rbcL*	375	CTT (L) → TTT (F)	C-U editing
*C. barometz*	*atpH*	75	TCA (S) → TTA (L)	C-U editing
	*psaB*	255	TCA (S) → TTA (L)	C-U editing
	*psbB*	238	TCA (S) → TTA (L)	C-U editing
	*psbE*	30	CCG (P) → CTG (L)	C-U editing
	*psbL*	31	CCA (P) → CTA (L)	C-U editing

**Fig. 8:**
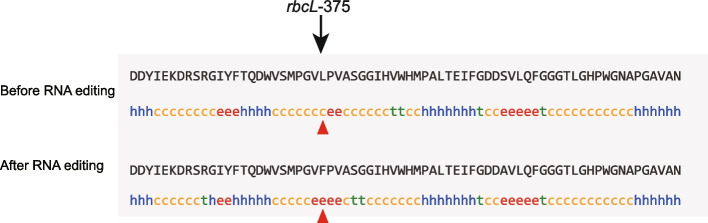
Protein secondary structure of *rbcL* before and after editing of *A. spinulosa*. The arrow refers to the editing sites. Hh refers to α-helix, Cc refers to random coil, Ee refers to extended strand, Tt refers to β-fold

The secondary structure of the protein encoded gene was analyzed by further studying the site where RNA editing took place. In *A. spinulosa*, only the 375L secondary structure of *rbcL* gene changed from random coil (Cc) to extended strand (Ee) in positive selection sites for RNA editing (Fig. 8). No significant secondary structure changes were observed in the positive selection sites for RNA editing in *S. brunoniana*, *A. latebrosa*, and *C. barometz*.

### Chloroplast gene expression level

We calculated the gene expression levels of 29, 38, 17, and 22 protein-coding genes extracted from the transcriptome of *S. brunoniana*, *A. latebrosa*, *A. spinulosa*, and *C. barometz*. The results indicated that the highly expressed chloroplast genes in *S. brunoniana* were *psbA, rbcL, psbC, psbE* and *rps14* (Table 8). The highly expressed chloroplast genes in *A. latebrosa* were *psbA, rbcL, psbE, psbC* and *psbD* (Table 9). A significant expression of *psaA*, *psaB*, *rbcL*, *psbH*, and *psbC* genes was found in *A. spinulosa*. (Table 10). The chloroplast genes with high expression were *psbA, psbC, rbcL, rps14* and *rpl21* in *C. barometz* (Table 11). In conclusion, the highly expressed genes in these four species mainly belonged to photosystem I (*psa*-), photosystem II (*psb-*) and Rubisco large subunit (*rbcL*).

**Table 8:** Expression levels of chloroplast protein coding genes of *S. brunoniana*

Genes	Length(bp)	TPM	Genes	Length(bp)	TPM
*psbA*	1062	567,132.49	*atpA*	1524	2,546.76
*rbcL*	1428	116,345.85	*petA*	966	1,861.93
*psbC*	1386	44,193.70	*rpl20*	360	1,684.19
*psbE*	252	39,039.78	*rpl21*	357	1,674.10
*rps14*	303	35,122.70	*rps4*	624	1,355.35
*psbD*	1062	24,799.48	*ycf4*	555	1,052.35
*psbH*	225	22,381.03	*ndhJ*	522	921.94
*psaA*	2253	19,313.48	*ndhK*	756	671.44
*psaB*	2205	16,618.43	*ndhF*	2230	518.65
*psbB*	1527	13,192.74	*accD*	933	397.83
*rps18*	228	12,406.36	*rps11*	393	369.13
*atpE*	399	5,634.71	*rps12*	822	346.15
*atpB*	1482	5,090.72	*ndhC*	363	241.91
*petB*	1454	4,753.27	*rpoA*	1017	120.10
*petD*	1210	4,509.87

**Table 9:** Expression levels of chloroplast protein coding genes of *A. latebrosa*

Genes	Length(bp)	TPM	Genes	Length(bp)	TPM
*psbA*	1060	406,795.52	*rps14*	303	4,476.05
*rbcL*	1428	124,600.11	*petB*	1453	4,348.29
*psbE*	252	54,691.27	*petA*	966	3,754.15
*psbC*	1413	45,358.36	*rps4*	624	3,686.01
*psbD*	1146	30,721.06	*ndhJ*	522	2,717.39
*psaA*	2253	21,536.23	*atpF*	1227	2,600.65
*psbH*	225	19,239.92	*rps2*	753	2,386.48
*psaB*	2205	19,092.32	*accD*	933	2,382.20
*psbB*	1527	16,640.03	*chlB*	1542	2,076.96
*atpE*	399	14,469.60	*ycf4*	555	1,932.88
*rps18*	228	13,780.79	*ndhK*	756	1,831.89
*atpB*	1482	12,423.85	*ndhC*	363	1,224.51
*rpl33*	201	10,678.02	*ycf66*	698	1,204.84
*chlL*	882	6,596.42	*rpoA*	1017	1,127.75
*atpI*	747	6,459.10	*rps8*	396	1,106.48
*petD*	1199	5,894.89	*rps11*	393	1,011.80
*rpl21*	357	5,839.69	*cemA*	1521	761.00
*chlN*	1395	5,662.78	*ndhF*	2232	726.96
*atpA*	1524	4,654.68	*ycf3*	1877	588.82

**Table 10:** Expression levels of chloroplast protein coding genes of *A. spinulosa*

Genes	Length(bp)	TPM	Genes	Length(bp)	TPM
*psaA*	2253	150,148.15	*atpH*	246	20,777.00
*psaB*	2205	98,663.00	*psbB*	1527	15,082.32
*rbcL*	1428	93,230.64	*rps4*	624	14,570.58
*psbH*	225	67,622.48	*ndhJ*	522	10,502.05
*psbC*	1386	41,350.05	*ndhC*	363	8,419.91
*psbD*	1062	38,885.96	*atpB*	1482	6,700.85
*rps14*	303	27,415.06	*ndhK*	756	6,699.99
*psbE*	252	25,158.43	*atpE*	399	1,769.65
*rpl21*	357	24,144.02

**Table 11:** Expression levels of chloroplast protein coding genes of *C. barometz*

Genes	Length(bp)	TPM	Genes	Length(bp)	TPM
*psbA*	1062	188,152.46	*petA*	966	14,397.57
*psbC*	1386	61,840.38	*psbE*	252	12,198.55
*rbcL*	1428	56,044.64	*ndhF*	2229	10,529.56
*rps14*	303	52,477.13	*rps16*	1030	8,634.18
*rpl21*	357	46,429.28	*atpI*	747	8,237.63
*psaA*	2253	44,820.67	*atpA*	1524	6,856.81
*psbD*	1062	41,007.59	*rps2*	753	6,386.53
*psaB*	2205	37,352.74	*chlN*	1395	5,749.78
*psbH*	225	26,603.34	*ndhG*	594	4,915.26
*chlB*	1542	24,494.99	*petB*	1445	4,467.26
*psbB*	1527	15,069.39	*atpF*	1251	2,483.48

## Discussion

### Transcriptome sequencing of different organs

This study combined the PacBio SMRT-Seq and Illumina RNA-Seq to analyze the full-length transcripts and genes expression patterns of three organs of *S. brunoniana* and *A. latebrosa*. Transcriptome data reflect the number and types of genes expressed in different organs and reveal potential metabolic pathways and genetic mechanisms [45]. Transcriptome sequencing is an effective and feasible method to generate gene sequence data, and its large number of cDNA sequences provide useful resources for genome and genetic studies [46]. SMRT sequencing has become the most reliable and effective strategy for full-length transcriptome analysis, especially for non-model plant species without reference genome sequences [46]. At present, both *S. brunoniana* and *A. latebrosa* do not have reference genomes, so it is efficient to understand their gene information through transcriptome sequencing. In this study, we ultimately obtained 11,625, 14,391, and 10,099 unigenes for the root, rachis, and pinna of *S. brunoniana*, respectively. Similarly, a total of 13,028, 11,431, and 12,144 unigenes were obtained from the root, rachis, and pinna of *A. latebrosa*. respectively. Illumina sequencing has the advantages of high accuracy of single base, which PacBio SMRT sequencing can provides ultra-long sequence reading [47]. In this study, the two methods were combined to sequence different organs of *S. brunoniana* and *A. latebrosa*. The overall genetic information was sufficient and the sequencing quality was high.

### Gene annotation and gene structure

Full-length transcriptome sequencing based on PacBio sequencing can significantly optimize gene annotation [48], providing high-quality genetic and molecular information for *S. brunoniana* and *A. latebrosa*. In addition, in this study, a large number of TFs were identified in the transcriptome of *S. brunoniana* and *A. latebrosa*, including AP2/ ERF-ERf, Tify, C3H and MYB. These TFs play a crucial role in plant stress response. Within the AP2/ERF superfamily, AP2 transcription factors are primarily involved in the regulation of development, while ERF proteins are primarily respond to environmental stress response [49]. TIFY family regulates plant defense activities through Jasmonic acid induction, and is part of the biological negative feedback and signal transduction pathway, which can control Jasmonate ZIM domain protein levels under jasmonic acid and environmental stress [50, 51]. Therefore, these TFs provide molecular data for the study of plant stress tolerance.

Studies have shown that lncRNAs involved in a wide range of biological processes including gene transcription and post-transcriptional modification, epigenetic level regulation of gene expression, genomic imprinting, chromatin remodeling, transcriptional activation, transcriptional interference and cell cycle [52]. For instance, there was a significant alteration observed in the expression of lncRNA (At5NC056820) in response to drought stress in *Arabidopsis thaliana* [53]. This suggests that lncRNAs might participate in the response to drought stress. Furthermore, some lncRNAs related to growth and wood characteristics have been found in trees (*Populus tomentosa*) [54, 55], which could provide a valuable genetic background for the subsequent exploration of the growth of dendritic plants. Thus, it can be inferred that a vast number of lncRNAs in *S. brunoniana* and *A. latebrosa* are involved in regulating their growth and adapting to the environment.

Organisms must adapt to environmental changes to survive, and a certain degree of stress caused by environmental fluctuations is the necessary starting point for each adaptation [56]. SSRs act as an evolutionary adjustment knob to provide evolutionary advantages of rapid adaptation to new environments [57]. In the transcriptome of *S. brunoniana*, 4,294, 6,520 and 3,926 SSRs were identified in the root, rachis and pinna, respectively. Totally, 4,918 (root), 5,350 (rachis) and 5,292 (pinna) SSRs were identified from the transcriptomes of three organs of *A. latebrosa*. The presence of abundant SSRs within the genomes of both *S. brunoniana* and *A. latebrosa* indicates SSRs function in facilitating their adaptation to the surrounding environment. Earlier studies speculated that eukaryotes with more DNA repeats might provide a molecular device for faster adaptation to environmental pressures [57, 58]. SSR can be used as "tuning knobs" to gradually regulate gene expression or other functions by repeating copy number within the range allowed by the discrete number of repeat sequences. The greater the number of repeats, the weaker the effect of single repeats and the better the tuning effect [56]. Therefore, the copy number of SSR repeats in turn affects the phenotype [56]. In addition, many SSR sequences are considered to be hot spots for recombination [59], especially dinucleotide repeats, which have high affinity for recombination enzymes and are preferred sites for recombination [60]. Some SSR sequences directly affect recombination by affecting DNA structure [58]. In addition, as an important molecular marker, SSR plays an important role in the analysis of germplasm resources identification, genetic diversity and genetic relationship, and has been widely used in systematic geography. The abundant SSR resources obtained in this study can provide high-quality molecular resources for the follow-up study of *S. brunoniana* and *A. latebrosa* genetic geography and environmental adaptation analysis.

### Differential expression between organs

We observed the highest number of DEGs between the pinna and the root. Further enrichment analysis revealed that the DEGs in different organs showed distinct patterns. Specifically, the DEGs in the pinna were significantly enriched in the processes of photosynthesis and photosystem, which strongly supported the notion that leaves are the main sites of plant photosynthesis. Moreover, an abundance of DEGs in the root and rachis were found to be involved in the plant secondary metabolic pathway. When considering the expression levels of genes associated with this pathway, it became evident that active secondary metabolic processes occur in the rachis and root.

### Genes and pathways associated with environmental adaptation

The adaptability of plants to environmental stress is a universal ecological behavior in nature. Secondary metabolism is a key component of the interaction between plants and the environment to adapt to biological and abiotic stress conditions [61]. Phenylpropanoid synthesis pathway is the main synthetic contact center for the production of many plant metabolites, so it is one of the most critical metabolic pathways for plants to adapt to the environment [23].

Lignin synthesis is closely related to the erect rhizome of tree ferns. Lignin, provides an upright rigid structure for vascular plants and strengthens the cell walls of water-conducting cells [22]. Therefore, in order to investigate the development of woody trunks in tree ferns, we conducted an analysis of the lignin pathway. Two lignin synthesis pathways were identified in *S. brunoniana* transcriptome. The one was H-lignin synthesis pathway, and the other was G-lignin synthesis pathway. H and G lignin are the basis of all vascular plants [22]. Lignin is critical for the development of plants, and its biosynthesis may be stimulated by photocompetition [21]. In addition, lignin acts as a waterproofing of cell walls, allowing water and solutes to be transported through the vascular system and induced as a defense response to protect plants from pathogens after injury or pathogen attack [62]. The genes and pathways related to lignin synthesis identified in this study provide data support for future studies on large tree ferns.

WRKY transcription factors are one of the largest TFs families in plants and are part of signal networks that regulate many plant processes and involve in plant growth, immunity, and regulatory signaling networks [26]. The WRKY gene family was generated through gene duplication during evolution [27], performing pivotal functions in regulating of plant physiological development and orchestrating stress responses. Based on the research of Eulgem et al. [43], 85 WRKY transcription factors, including three types, were identified in the transcriptomes of *A. latebrosa*. Group I is the ancestor type of WRKY gene [26, 63], and members of this family have been identified in a variety of plants. For example, 74 WRKY members were identified in Arabidopsis, and 75 were identified in *Medicago truncatula*. A total of 104 were identified in *Populus trichocarpa* [26, 63]. In response to different biological stresses, WRKY transcription factors involve in the activation of Salicylic acid, Jasmonic acid and Ethylene signaling pathways, and then change the transcription levels of related genes to help plant defense mechanisms to counter pathogen attacks [64, 65]. In addition, WRKY is part of a complex hormonal signaling network. It can participate in plant immunity by regulating Jasmonic acid and Salicylic acid, and affect plant development by regulating Auxin and Cytokinin [27]. Different plant hormones have different effects on WRKY transcription factors. For example, Rushton et al. [66] showed that WRKY regulates stomatal closure by mediating Abscisic acid expression to respond to drought stress in plants. Because WRKY is a large transcription factor family, it contains abundant gene members. Same as other large gene families, the problem of functional redundancy complicates the determination of the role of individual WRKY proteins [43]. The WRKY transcription factor family information obtained in this study can provide rich molecular resources for the subsequent analysis of WRKY protein functions in tree ferns.

Plant hormones coordinate the complex plant development process by integrating environmental stimuli [67] to extensively participate in various physiological activities of plants. Based on KEGG annotation, a total of 463 unigenes were identified to be involved in hormone signal transduction. *GID1* gene encoding Gibberellin receptor and *JAZ* gene encoding Jasmonic acid ZIM domain protein were highly expressed in *A. latebrosa*. GID1 was first discovered in rice (*OsGID1* gene) [68]. Gibberellin is a diterpenoid plant hormone, which regulates many physiological activities of plants. Little et al. [69] demonstrated that in *Pinus sylvestris* and *Picea Glauca*, gibberellin stimulates the activity of the proximal apical meristem of new plants. In addition, gibberellin is also one of the important factors determining plant height [70]. JAZ proteins belong to the plant specific TIFY family [71]. JAZ repressors play a central role in the jasmonic acid-triggered signaling cascade [72].

### Adaptive evolution of RNA editing sites

In this study, four, five, six and five positive selection sites of *S. brunoniana*, *A. latebrosa*, *A. spinulosa*, and *C. barometz* were identified as RNA editing sites, and the remaining RNA editing sites were purified selection sites. These positive selection sites were mainly located in *atpH, psbB, psbE, psbL, psaB* and *rbcL* genes. Overall, relatively few positive selection sites for RNA editing occurred in these 18 functionally important chloroplast genes. By comparing the evolution of RNA editing in the chloroplasts of relict plant *Ginkgo biloba*, we found that purified selection constituted the main evolutionary force of RNA editing sites of essential genes in the chloroplasts of *G. biloba*, such as partial *psa-* and *psb-* genes [41, 73]. Therefore, we hypothesized that RNA editing sites were more likely to be purified selection among functionally important essential genes. Since RNA editing may be a post-transcriptional regulatory process of ancient genes, it is also part of an evolutionary model with different evolutionary directions [74]. Therefore, we further speculated that editing sites in each gene may undergo different evolutionary paths, depending on whether the edited codon is important for protein executive function [73]. Plants influence final protein products through RNA editing, in which RNA editing may provide an initial selective advantage that facilitates fixation and further propagation in chloroplast genes [37]. In addition, our study showed that RNA editing can affect the secondary structure of proteins, such as the transformation of 375L in the *rbcL* gene from random curl (Cc) to extended chain (Ee), but not all RNA editing sites alter the secondary structure of proteins.

All RNA editing types identified in this study are C-U editing, which is widely found in chloroplast genes of plants [36]. Conversion of C residues to U helps maintain protein conserved or create the correct open reading framework [75]. In the chloroplasts of *G. biloba*, all RNA editing sites were also C-U conversion [41, 73]. In addition, there is an interesting relationship between plastid RNA editing and genetic code, and studies have shown that most plastid editing events affect the position of the second codon [41, 73]. In this study, RNA editing events also mainly occurred at the second codon location.

At present, there is no unified conclusion on the evolutionary history of RNA editing, and the controversy focuses on whether its origin is merely a historical accident or an inevitable evolutionary innovation [39]. Several evolutionary ideas have been proposed, including random genetic drift [37], adaptive editing [38] and enzyme mutation [39, 40]. With more data and more extensive analysis, combined with the results of this study, we tend to agree that the RNA editing event itself originates from a neutral event, however it may confer some adaptation on the organelle genome in subsequent development. Otherwise, it is inconceivable that such mutations could have remained irreparably and unchangeably in the organelle genome of land plants over the course of over 400 million years of evolution [39].

## Conclusions

This study investigated three organs of *S. brunoniana* and *A. latebrosa* using NGS and PacBio SMRT sequencing. Transcriptome data were used to mine chloroplast genes, identify RNA editing sites, and calculate gene expression levels. From the NGS and PacBio SMRT sequencing, we obtained high-quality unigenes from the roots, rachis, and pinna of *S. brunoniana* and *A. latebrosa*, respectively. Furthermore, TFs, SSRs, and lncRNAs were identified. Gene expression patterns and DEGs were also analyzed. KEGG enrichment analysis revealed that unigenes with higher expression had special roles in environmental stress response and adversity adaptation. Additionally, two lignin synthesis pathways (H-lignin and G-lignin) were found in *S. brunoniana*. Moreover, 16 positively selected sites were identified from 18 chloroplast protein-coding genes in four tree ferns, only a small fraction of which underwent RNA editing. We hypothesized that RNA editing sites were more likely to be purified selection among functionally important essential genes. RNA editing affected the protein secondary structure of the *rbcL* gene. The calculation of gene expression levels of chloroplast protein-coding genes showed higher expression levels of genes related to chloroplast photosynthetic systems. This study has enriched the gene information of tree ferns and deepened our understanding of their gene structure, gene expression levels, and environmental adaptability. High-quality transcriptome data sets of *S. brunoniana* and *A. latebrosa* have been constructed, providing abundant molecular resources for fern research. The mining of chloroplast genes in transcriptome data helps us to comprehensively understand the expression levels of chloroplast genes and RNA editing events. With the rich gene data obtained in this study, future research can be conducted in-depth physiological analysis of these genes involved in environmental adaptation to verify their specific functions in response to environmental stress.

## Materials and methods

### Plant materials and RNA extraction

Our research complies with the laws of the People’s Republic of China. The voucher specimens of *S. brunonian*a (voucher number: ChenAB201905) and *A. latebrosa* (voucher number: ChenAL201905) were identified by Qing Chen, and stored at the Herbarium of Sun Yat-sen University.

The fresh root, rachis, and pinna of *S. brunoniana* and *A. latebrosa* were collected at 884 m (19°4′34" N, 109°8′46"E) and 920 m (19°4′5"N, 109°8′29"E) in Bawangling, Hainan Province, China, respectively. The samples were washed immediately and dried up before being immersed in RNAlater solution (BioTeke, Shanghai, China). The samples were preserved at -20°C until RNA extraction. RNeasy Plus Mini Kit (Qiagen) was used to extract total RNA from the root, rachis and pinna of *S. brunoniana* and *A. latebrosa*. Nanodrop 2000 spectrophotometer (Thermo Fisher Scientific, MA, USA), Qubit 2.0 fluorometer (Thermo Fisher Scientific, MA, USA) and Agilent 2100 Bioanalyzer (Agilent Technologies, CA, USA) were used to detect the samples. High quality RNA was used for cDNA synthesis and library construction.

### Illumina library preparation, sequencing and de novo assembly

The NEBNext Ultra RNA Library Prep Kit for Illumina (New England Biolabs, Ipswich, MA, United States) was used to prepare the Illumina library. Sequencing was performed on the Illumina NovaSeq platform (Illumina, San Diego, CA, USA), generating paired-end (PE) reads. Then the following raw reads were filtered: the reads containing adapter, the reads with more than 10% unknown bases, and the reads with more than 50% low-quality bases (QPhred ≤ 20). The clean reads generated from each organ were used for self-assembly by Trinity v2.4.0 [76], with parameters set as min_kmer_cov: 3 and other default parameters. Subsequently, the de novo assembly sequences were clustered to obtain the unigenes by Corset v1.05 [77].

### PacBio library preparation, sequencing and preprocessing

Total RNA for each of the three organs was separately used to construct libraries according to the PacBio Isoform Sequencing (Iso-Seq) experimental protocol. These PacBio libraries were sequenced on the PacBio Sequel II platform (Pacific Biosciences, Menlo Park, CA, USA). Subreads were obtained by removing the connector and the data with a length less than 50 bp. The subreads file was processed using the Circular Consensus Sequence (CCS) algorithm in SMRTlink 7.0 software (http://www.pacb.com/products-and-services/analytical-sofware/smrt-analysis/), with the following parameters: –min_length 50, –max_length 15,000, –min_passes 1. Arrow was used to calibrate the consensus sequence [78]. To further improving the sequencing accuracy and validate the polished consensus sequence with second-generation data, LoRDEC [79] software was employed with following parameters: -k 23, -s 3. Finally, CD-HIT v. 4.6.8 [80] was used to cluster the corrected transcript sequences based on 95% similarity, using the following parameters as: -c 0.95, -T 6, -G 0, -aL 0.00, -aS 0.99, -AS 30.

### Functional annotation of transcripts

The final obtained non-redundant transcript sequences were functionally annotated using the following databases: NCBI Nr database, KOG database, Swiss-Prot database, KEGG database, NCBI Nt database, GO database and Pfam database. The first four databases annotations were performed using DIAMOND v. 0.8.36 with an E-value threshold of 1.0 × 10^−5^ [81]. We used ncbi-blast-2.7.1 + [82] with an E-value threshold of 1.0 × 10^−5^ and Hmmscan of the HMMER 3.1 package [83] (http://hmmer.org/) for NCBI Nt database annotation and Pfam database annotation.

### Prediction of CDS, TFs, LncRNA and SSR

ANGEL v. 2.4 (https://github.com/PacificBiosciences/ANGEL) was used to predict the CDSs from cDNAs. The parameter setting was: –min_angel_aa_length 50, and the remaining options were the default. iTAK V1.7a [84] was used to predict the plant TFs. The parameters were set as follows: -f 3F. To obtain a set of high confidence lncRNAs, CNCI v. 2 [85], CPC v. 0.9 [86], and PLEK v. 1.2 [87] were employed to lncRNA prediction through screening coding potential. Unigenes from Iso-Seq were selected for SSR analysis using MISA v. l.0 [88] with the following minimum repeat times: mono-10, di-6, tri-5, tetra-5, penta-5, and hexa-5.

### Gene Expression quantification and differentially expressed gene analysis

RSEM v1.3.0 [89] software was used to count the readcount value of each gene in each organ, and then FPKM was adopted to determine the expression level of each unigene. DEGseq v. 1.12.0 software [90] was used for differential expression analysis of genes. Unigenes with *q* value < 0.005 and |log_2_ (fold change) |> 1 were considered to be the DEGs.

### Gene family analysis

Based on functional annotations from publicly available databases (Nr, Swiss-Prot, Pfam, and KOG), the pathview package [91] in R software was used to characterize the lignin synthesis pathway of *S. brunoniana*, and the gene expression level involved in related pathways were drawn. Based on the transcription factor identification results, the WRKY transcription factor family members were screened. CD-HIT [80] was used to de-redundancy the sequence with a 95% threshold. Protein domains were predicted using HMMER (http://hmmer.org/) [92], E value was set to 1 × 10^–5^, and domain information was visualized using TBtools V1.6 [93]. Related genes involved in hormone signal transduction pathway [94–96] of *A. latebrosa* were identified by KEGG enrichment results. The pathview package [91] in R software was used to characterize the hormone signal transduction pathway, and the expression level of related genes was drawn.

One of the *4CL* unigenes of *S. brunoniana* and *JAZ* unigenes of *A. latebrosa* were utilized to validate the expression through qRT-PCR. In essence, total RNA from the root, rachis, and pinna was extracted as previously described and subsequently reverse-transcribed into cDNA templates using the HiScript III RT SuperMix for qPCR Kit (Vazyme, Nanjing, China). The primer pairs were designed using Primer3Plus [97]. The qRT-PCR assay was performed conducted in triplicate utilizing the ChamQ SYBR Color qPCR Master Mix Kit (Vazyme, Nanjing, China). The PCR procedures comprised of an initial step at 95°C for 30s, followed by a step at 95°C for 10s, 60°C 30s, for 40 replicates verified by the standard melting curve. The actin unigene was used as the reference gene. The relative expression of *4CL* unigene and *JAZ* unigene were calculated by 2^–ΔΔCt^ method [98].

### Extraction of chloroplast gene from transcriptome

Beside the transcriptome data of *S. brunoniana* and *A. latebrosa* in this study, we also obtained the same type data of other two ferns from our research team (*A. spinulosa* (SRX11010895, SRX11012686, SRX11012685, SRX11012684) and *C. barometz*). Combined with Illumina sequencing data and PacBio SMRT sequencing data of *S. brunoniana*, *A. latebrosa*, *A. spinulosa*, and *C. barometz*, chloroplast genes in transcriptome data were mined.

The chloroplast genomes of *S. brunoniana*, *A. latebrosa*, *A. spinulosa*, and *C. barometz* were obtained from NCBI database, with the accessions were NC_051561, MW620065, NC_012818, and NC_037893, respectively. Chloroplast genome was set as reference genome. Bowtie2 v2.4.4 [99] software was used to compare Illumina sequencing data to the reference genome and extract relevant sequences of chloroplast genes. The parameters were set as follows: Q - sensitive - end - to - end. For the extraction of chloroplast genes from the Pacbio sequencing data, the BLASR V5.1 [100] was used to align the PacBio SMRT data to the reference genome, and the parameters were set as: – bestn 1 -m 1 –minMatch 15 to obtain the chloroplast gene sequence. Finally, Unicycler V0.4.8 [101] software was used to assemble the chloroplast gene sequences extracted from both the Illumina and PacBio SMRT sequencing data, employing the default parameters.

### Phylogenetic analysis

In order to further studying the adaptive evolution and RNA editing of chloroplast genes (especially photosynthetic system genes) in tree ferns, 18 chloroplast genes in ferns were selected for analysis. There were fifteen photosynthetic system genes (*atpH, psaA, psaB, psbB, psbC, psbD, psbE, psbF, psbH, psbI, psbK, psbL, psbT, psbZ,* and *rbcL*), two genetic system related genes (*rpl21, rpl 32*) and one other genes (*ycf12*).

A phylogenetic tree was constructed based on 18 protein-coding genes of 88 fern species from 33 families (Table S18). The data of *S. brunoniana*, *A. latebrosa*, *A. spinulosa*, and *C. barometz* were derived from sequences extracted in this study. Sequence data of other species were obtained from GenBank database of National Center for Biotechnology Information (NCBI) (https://www.ncbi.nlm.nih.gov/). Geneious V9.0.2 [102] was used to extract 18 protein-coding genes from another 84 species of ferns. According to the extracted genes, a gene set was constructed for each gene. MEGA7 [103] was used to perform ClustalW sequence alignment and manual correction on the gene set based on codon alignment, and the internal and terminal stop codons were deleted. Finally, a gene set consisting of 18 gene sequences was obtained. Maximum likelihood (ML) analysis of gene sets was performed using RAxML V8.2.12 [104]. Based on the GTRGAMMA model, 1,000 bootstrap replicates were conducted. Finally, the ML tree with the highest score (-f a) was selected. We employed FigTree v1.4.3 (http://tree.bio.ed.ac.uk/software/figtree/) to obtain the ML tree for further editing and beautification. The phylogenetic tree mainly provided tree files for adaptive evolution analysis of subsequent genes.

### Adaptive evolution of chloroplast genes

In this study, codeml program in PAML V4.9 [105] was used to run the site-specific ω models based on M1a-M2a and M7-M8, respectively. Empirical Bayes (BEB) [106], and then Likelihood ratio test (LRT) was used for significance analysis to screen positive selection sites.

### Analysis of RNA editing sites in chloroplast genes

The sites identified as positive selection were further explored whether RNA editing had occurred, count the types of RNA editing and analyze the effects on the secondary structure of proteins. Combined with transcriptome data and the Prep-CP [107] online tool (http://prep.unl.edu/) to predict the RNA editing sites of chloroplast genes, the parameter threshold was set to 0.8. The SOPMA method (https://npsa-prabi.ibcp.fr/cgi-bin/npsa_automat.pl?page=npsa_sopma.html) was used to predict the protein secondary structure at positive selection sites where RNA editing took place. Similarity threshold was set to 8.

### Analysis of chloroplast gene expression level

At present, transcriptome sequencing technology is developing rapidly, but the analysis of fern organelle gene expression level was often neglected. In this study, in order to obtain the expression levels of chloroplast protein coding genes of the pinna of *S. brunoniana*, *A. latebrosa*, *A. spinulosa*, and *C. barometz*, RSEM v1.3.3 software was used to set the chloroplast protein coding genes extracted from transcriptome data as the reference data set, and selected –paired end-sequencing parameters.

### Supplementary Information

**Additional file 1: Fig S1.** Statistics of Nr annotations of *S. brunoniana* (top ten). (a)Root. (b)Rachis. (C)Pinna. **Fig S2.** Structural prediction of three-organ full-length transcriptomes of *A. latebrosa*. (a) The quantity of lncRNAs in three organs. (b) Transcription factor family distribution (top ten). (c) Distribution of SSR motifs. The X axis represents the SSR motif units, i.e., the number of repeating bases. The Y axis represents the number of repetitions of the bases, where the specific repetition count corresponds to the colors mentioned in the legend. The Z axis represents the number of SSRs. **Fig S3.** Enrichment results of KEGG expression up-regulated genes in *S. brunoniana* pinna (compared with root and rachis) (top twenty). The significantly enriched pathways with corrected *p*-value (*q* value) < 0.05 were shown. Number indicates the size of the dot, describing the number of unigenes enriched in the pathway. The color bar represents the *q* value and indicates significance of the enrichment. **Fig S4.** Enrichment results of KEGG expression up-regulated genes in *S. brunoniana* root (compared with root and rachis) (top twenty). The significantly enriched pathways with corrected *p*-value (*q* value) < 0.05 were shown. Number indicates the size of the dot, describing the number of unigenes enriched in the pathway. The color bar represents the *q* value and indicates significance of the enrichment. **Fig S5.** Enrichment results of KEGG expression up-regulated genes in *S. brunoniana* rachis (compared with root and rachis) (top twenty). The significantly enriched pathways with corrected *p*-value (*q* value) < 0.05 were shown. Number indicates the size of the dot, describing the number of unigenes enriched in the pathway. The color bar represents the *q* value and indicates significance of the enrichment. **Fig S6.** Enrichment results of KEGG expression up-regulated genes in *A. latebrosa *pinna (compared with root and rachis) (top twenty). The significantly enriched pathways with corrected *p*-value (*q* value) < 0.05 were shown. Number indicates the size of the dot, describing the number of unigenes enriched in the pathway. The color bar represents the *q* value and indicates significance of the enrichment. **Fig S7.** Enrichment results of KEGG expression up-regulated genes in *A. latebrosa *root (compared with root and rachis) (top twenty). The significantly enriched pathways with corrected *p*-value (*q* value) < 0.05 were shown. Number indicates the size of the dot, describing the number of unigenes enriched in the pathway. The color bar represents the *q* value and indicates significance of the enrichment. **Fig S8.** Enrichment results of KEGG expression up-regulated genes in *A. latebrosa *rachis (compared with root and rachis) (top twenty). The significantly enriched pathways with corrected *p*-value (*q* value) < 0.05 were shown. Number indicates the size of the dot, describing the number of unigenes enriched in the pathway. The color bar represents the *q* value and indicates significance of the enrichment.

**Additional file 2: Table S1.** PacBio sequencing results of *S. brunoniana*. **Table S2.** PacBio sequencing results of *A. latebrosa*. **Table S3.** Illumina sequencing results of *S. brunoniana*. **Table S4.** Unigenes statistics of Illumina sequencing in *S. brunoniana*. **Table S5.** Illumina sequencing results of *A. latebrosa*. **Table S6.** Transcripts and unigenes statistics of *A. latebrosa*. **Table S7.** KEGG enrichment results of unigenes in *S. brunoniana*. **Table S8.** The expression characteristics of all unigenes in three organs of *S. brunoniana*. **Table S9.** The expression characteristics of all unigenes in three organs of *A. latebrosa*.

**Additional file 3: Table S10. **GO enrichment results of differentially expressed genes of *S. brunoniana* pinna and root.

**Additional file 4: Table S11.** GO enrichment results of differentially expressed genes of *S. brunoniana* root and rachis.

**Additional file 5: Table S12. **GO enrichment results of differentially expressed genes of S. brunoniana pinna and rachis.

**Additional file 6: Table S13**. GO enrichment results of differentially expressed genes of *A. latebrosa* pinna and root.

**Additional file 7: Table S14**. GO enrichment results of differentially expressed genes of *A. latebrosa* root and rachis.

**Additional file 8: Table S15**. GO enrichment results of differentially expressed genes of *A. latebrosa* pinna and rachis.

**Additional file 9: Table S16. **The results of qPR-PCR experiment and the primer pairs.

**Additional file 10: Table S17.** WRKY transcription factor family members of three organs in *A. latebrosa*, which were obtained by deleting unexpressed gene sequences and incomplete protein motifs, and eliminating redundancy with 95% similarity.

**Additional file 11: Table S18.** Statistics of the unigenes in the plant hormone signal transduction (ko04075) pathway of *A. latebrosa*.

**Additional file 12: Table S19.** Taxonomic information of 88 species.

## Data Availability

The raw reads of RNA-Seq of *S. brunoniana* were deposited in the SRA database (https://www.ncbi.nlm.nih.gov/bioproject) as follows: SRR24680814 (root); SRR24680815 (rachis); SRR24680816 (pinna). The subreads BAM file of Iso-Seq can retrieve from the SRA database: SRR24680811 (root); SRR24680812 (rachis); SRR24680813 (pinna). The raw reads of RNA-Seq of *A. latebrosa* were deposited in the SRA database as follows: SRR25182368 (root); SRR25182369 (rachis); SRR25182373 (pinna). The subreads BAM file of Iso-Seq can retrieve from the SRA database: SRR25182371 (root); SRR25182372 (rachis); SRR25182370 (pinna).

## Abbreviations

DEG: Differentially expressed gene
TF: Transcription factor
lncRNA: Long non-coding RNA
SSRs: Single sequence repeats
CCS: Circular consensus sequence
ML: Maximum likelihood
LRT: Likelihood ratio test
