# Supplementary material for: Characterization and analysis of multi-organ full-length transcriptomes in Sphaeropteris brunoniana and Alsophila latebrosa highlight secondary metabolism and chloroplast RNA editing pattern of tree ferns
Source: BMC Plant Biol. 2024 Jan 26;24:73. doi: 10.1186/s12870-024-04746-w (PMC10811885; doi:10.1186/s12870-024-04746-w)
Supplement: Supplementary file 1 — Additional file 1: Fig S1. Statistics of Nr annotations of S. brunoniana (top ten). (a)Root. (b)Rachis. (C)Pinna. Fig S2. Structural prediction of three-organ full-length transcriptomes of A. latebrosa. (a) The quantity of lncRNAs in three organs. (b) Transcription factor family distribution (top ten). (c) Distribution of SSR motifs. The X axis represents the SSR motif units, i.e., the number of repeating bases. The Y axis represents the number of repetitions of the bases, where the specific repetition count corresponds to the colors mentioned in the legend. The Z axis represents the number of SSRs. Fig S3. Enrichment results of KEGG expression up-regulated genes in S. brunoniana pinna (compared with root and rachis) (top twenty). The significantly enriched pathways with corrected p-value (q value) < 0.05 were shown. Number indicates the size of the dot, describing the number of unigenes enriched in the pathway. The color bar represents the q value and indicates significance of the enrichment. Fig S4. Enrichment results of KEGG expression up-regulated genes in S. brunoniana root (compared with root and rachis) (top twenty). The significantly enriched pathways with corrected p-value (q value) < 0.05 were shown. Number indicates the size of the dot, describing the number of unigenes enriched in the pathway. The color bar represents the q value and indicates significance of the enrichment. Fig S5. Enrichment results of KEGG expression up-regulated genes in S. brunoniana rachis (compared with root and rachis) (top twenty). The significantly enriched pathways with corrected p-value (q value) < 0.05 were shown. Number indicates the size of the dot, describing the number of unigenes enriched in the pathway. The color bar represents the q value and indicates significance of the enrichment. Fig S6. Enrichment results of KEGG expression up-regulated genes in A. latebrosa pinna (compared with root and rachis) (top twenty). The significantly enriched pathways with corrected p-v [file 12870_2024_4746_MOESM1_ESM.docx]

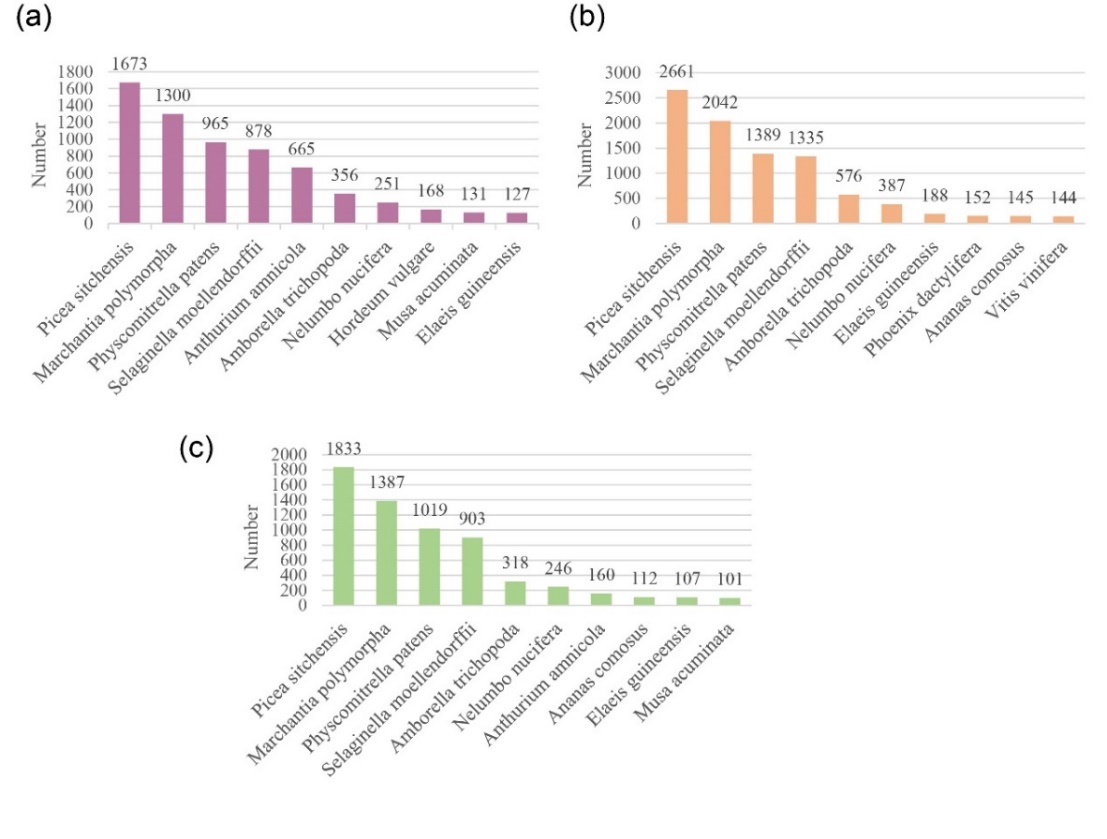


Fig. S1 Statistics of Nr annotations of *S. brunoniana* (top ten). (a)Root. (b)Rachis. (C)Pinna.


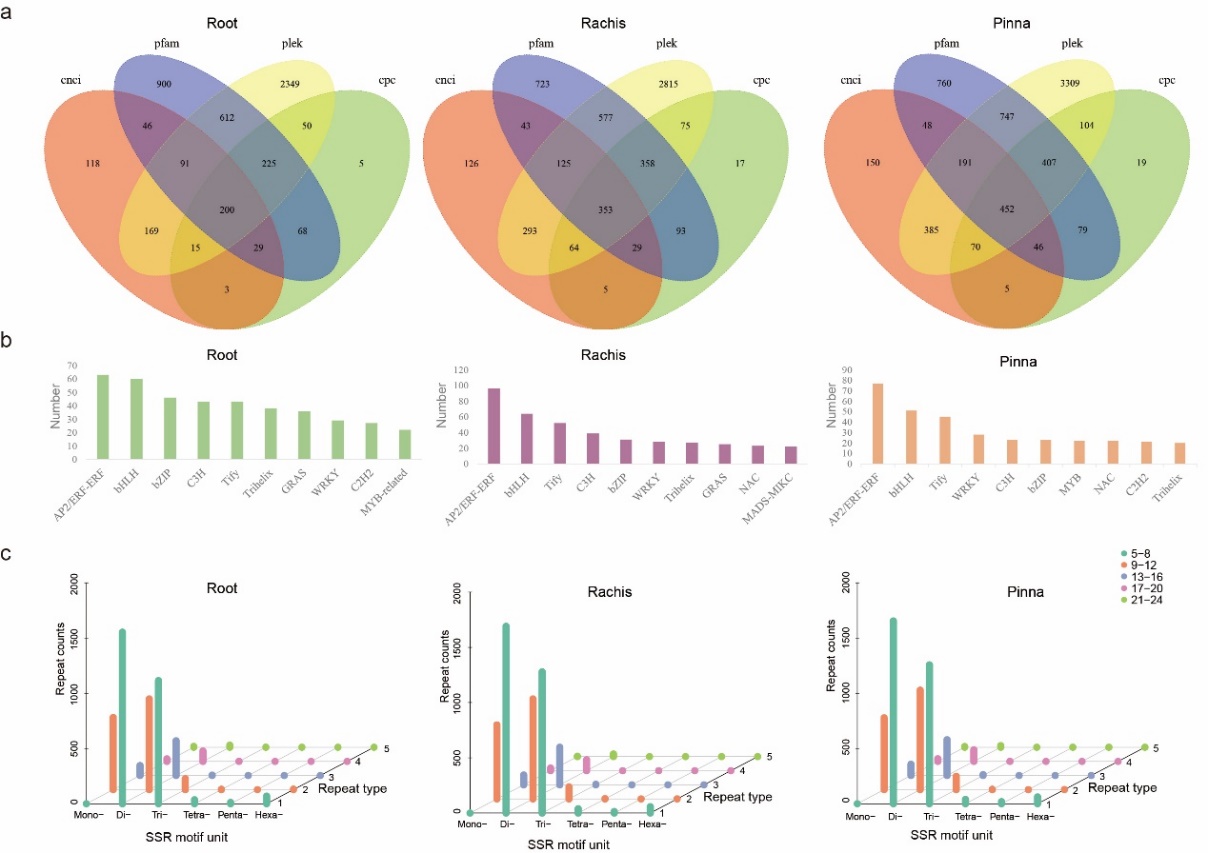


Fig. S2 Structural prediction of three-organ full-length transcriptomes of *A. latebrosa*. (a) The quantity of lncRNAs in three organs. (b) Transcription factor family distribution (top ten). (c) Distribution of SSR motifs. The X axis represents the SSR motif units, i.e., the number of repeating bases. The Y axis represents the number of repetitions of the bases, where the specific repetition count corresponds to the colors mentioned in the legend. The Z axis represents the number of SSRs.


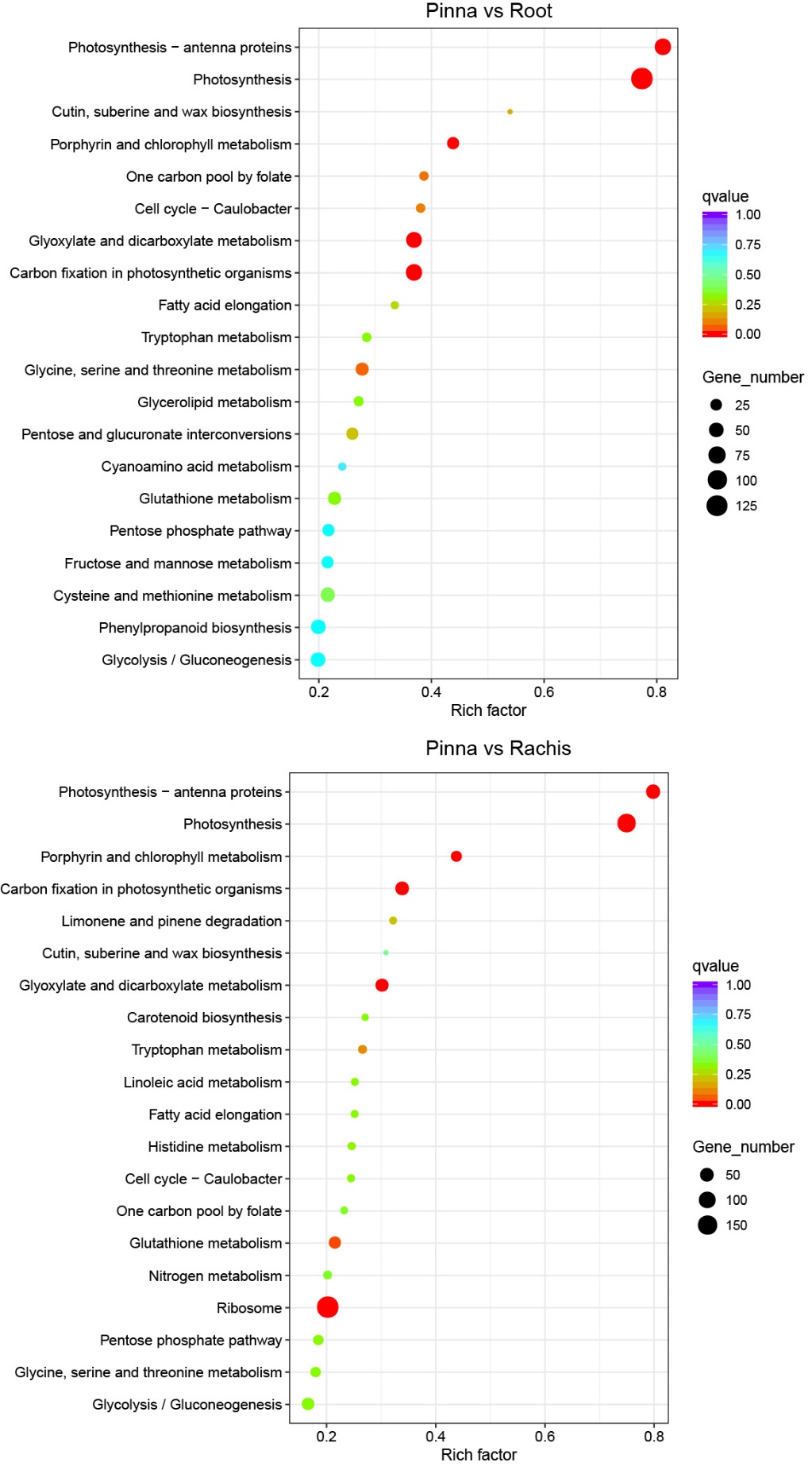


Fig. S3 Enrichment results of KEGG expression up-regulated genes in *S. brunoniana* pinna (compared with root and rachis) (top twenty). The significantly enriched pathways with corrected *p*-value (*q* value) < 0.05 were shown. Number indicates the size of the dot, describing the number of unigenes enriched in the pathway. The color bar represents the *q* value and indicates significance of the enrichment.


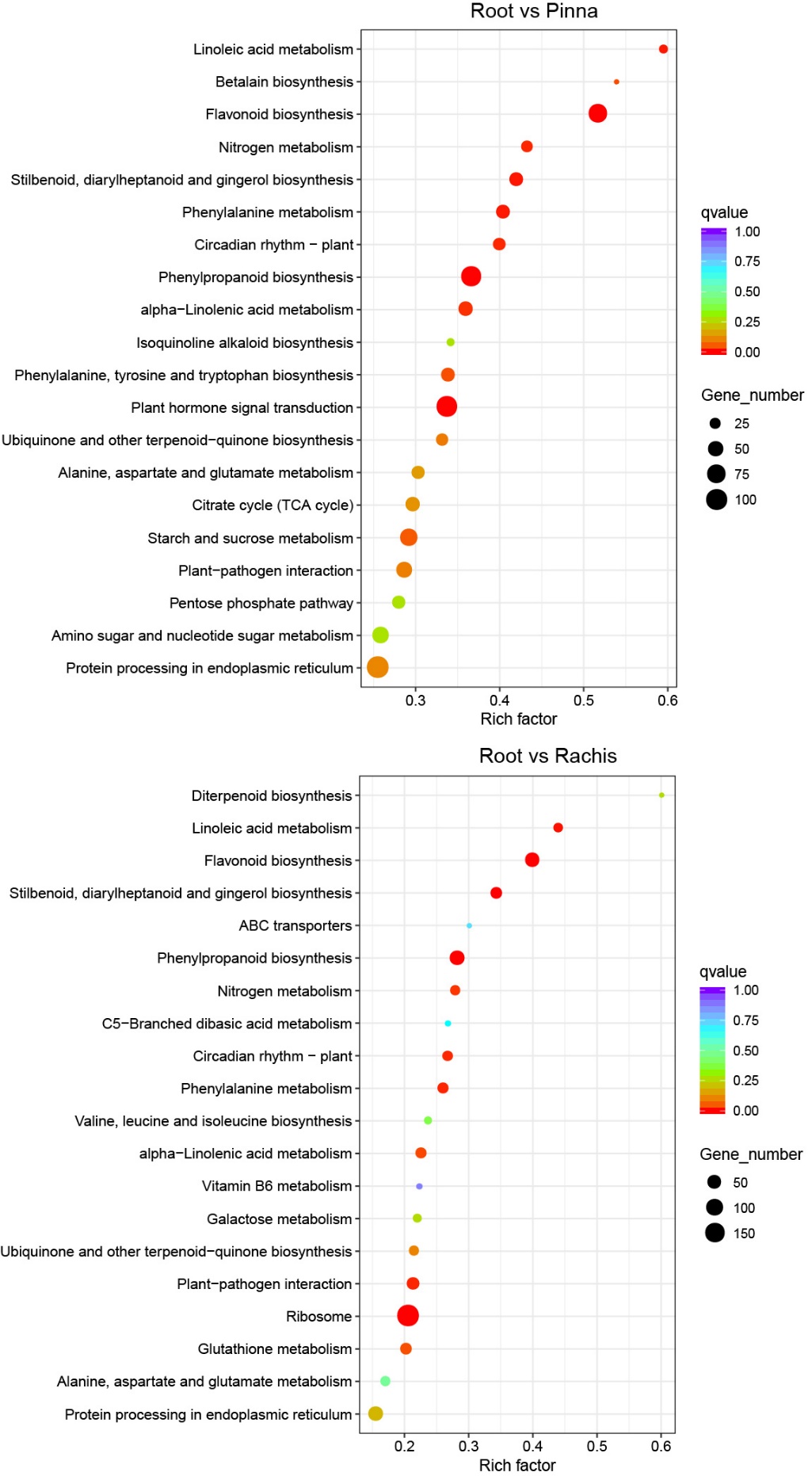


Fig. S4 Enrichment results of KEGG expression up-regulated genes in *S. brunoniana* root (compared with root and rachis) (top twenty). The significantly enriched pathways with corrected *p*-value (*q* value) < 0.05 were shown. Number indicates the size of the dot, describing the number of unigenes enriched in the pathway. The color bar represents the *q* value and indicates significance of the enrichment.


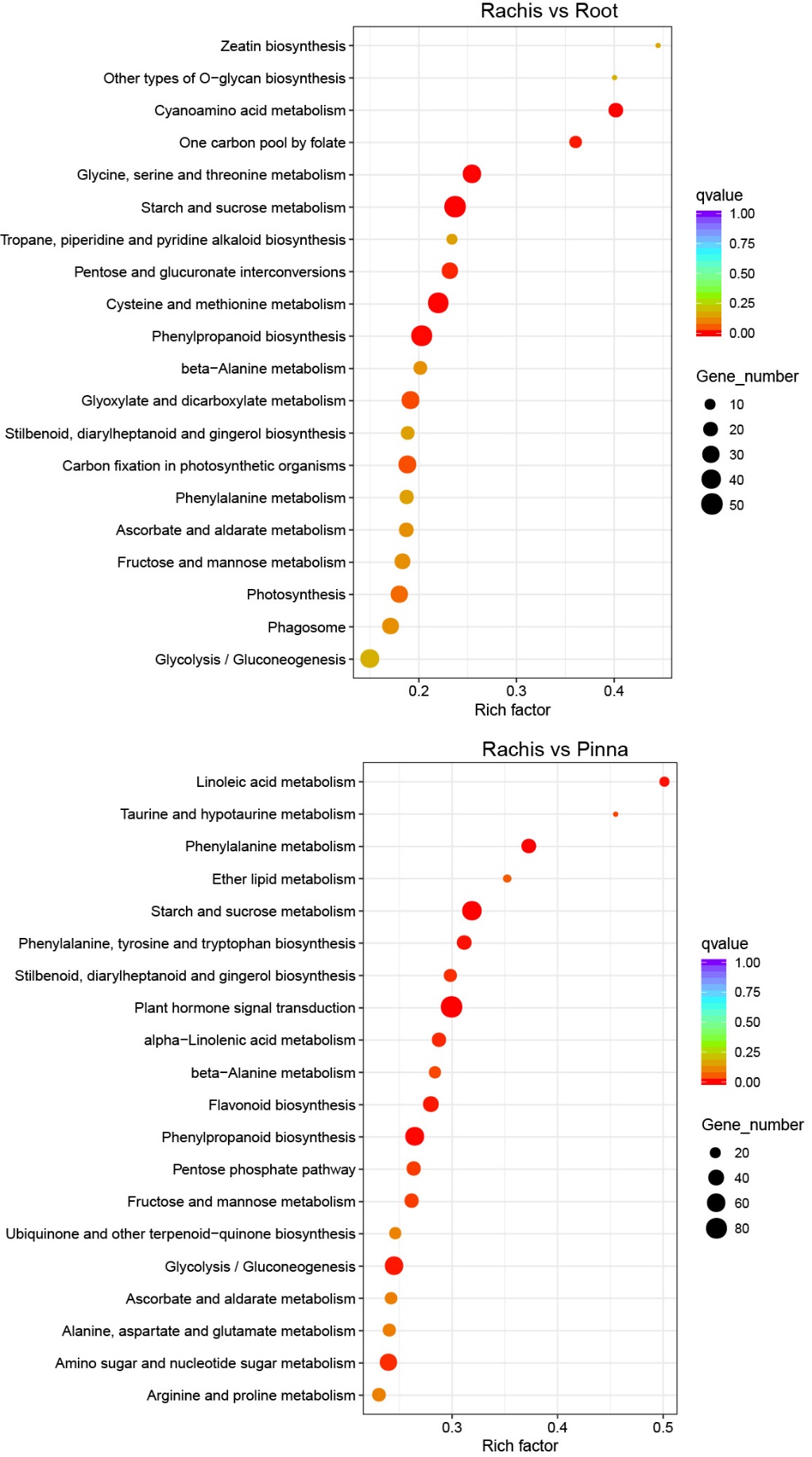


Fig. S5 Enrichment results of KEGG expression up-regulated genes in *S. brunoniana* rachis (compared with root and rachis) (top twenty). The significantly enriched pathways with corrected *p*-value (*q* value) < 0.05 were shown. Number indicates the size of the dot, describing the number of unigenes enriched in the pathway. The color bar represents the *q* value and indicates significance of the enrichment.


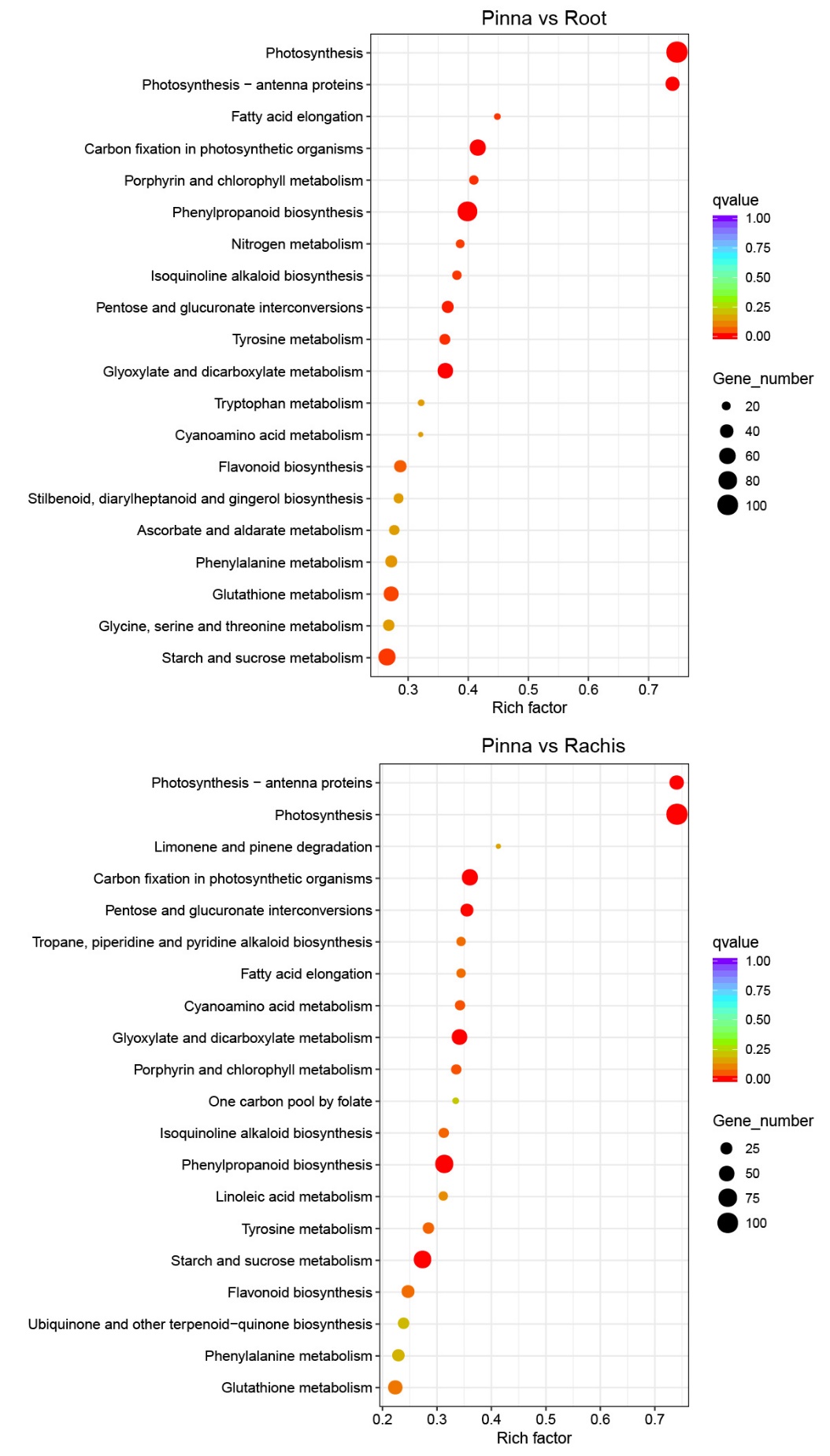


Fig. S6 Enrichment results of KEGG expression up-regulated genes in *A. latebrosa* pinna (compared with root and rachis) (top twenty). The significantly enriched pathways with corrected *p*-value (*q* value) < 0.05 were shown. Number indicates the size of the dot, describing the number of unigenes enriched in the pathway. The color bar represents the *q* value and indicates significance of the enrichment.


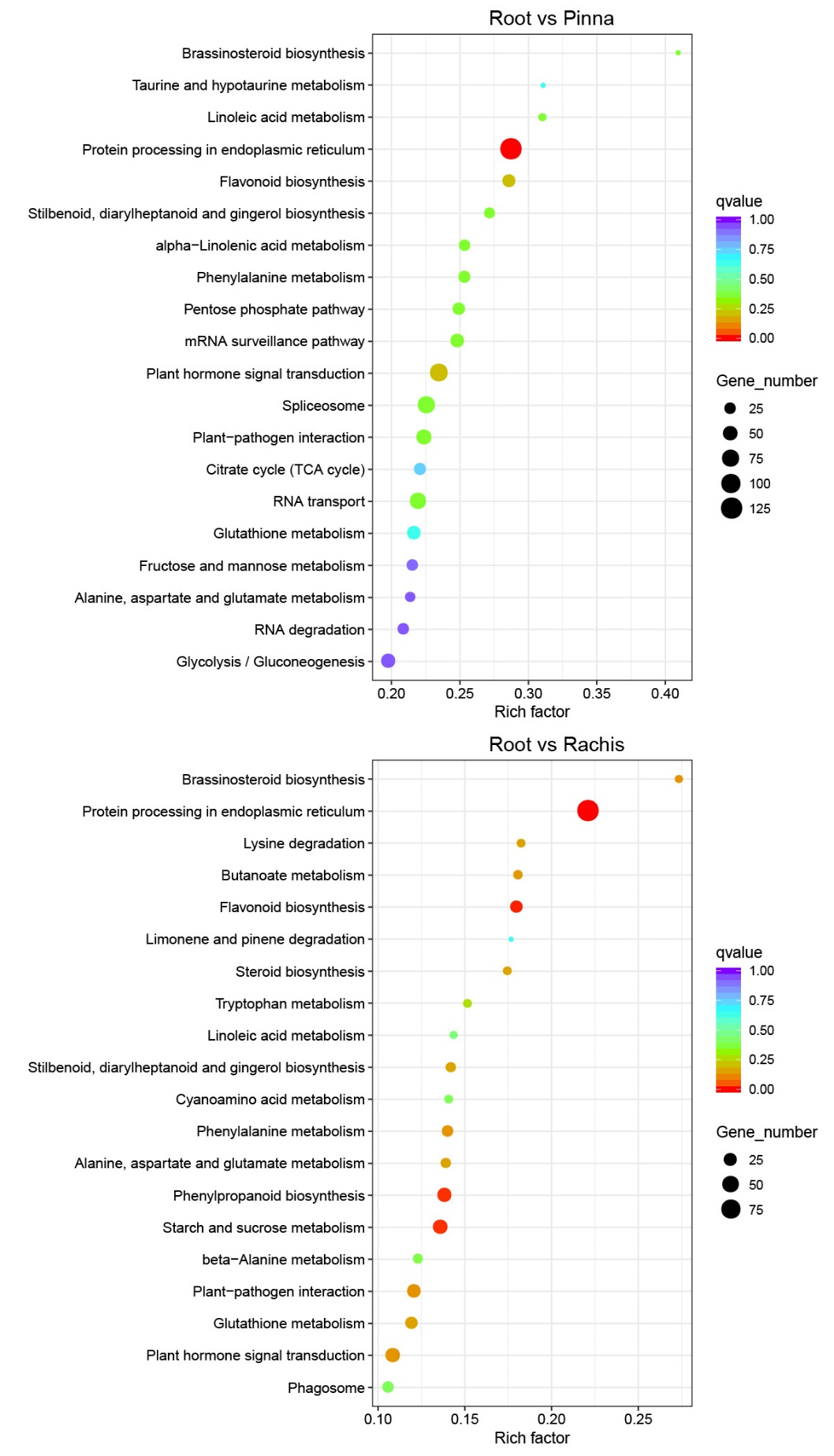


Fig. S7 Enrichment results of KEGG expression up-regulated genes in *A. latebrosa* root (compared with root and rachis) (top twenty). The significantly enriched pathways with corrected *p*-value (*q* value) < 0.05 were shown. Number indicates the size of the dot, describing the number of unigenes enriched in the pathway. The color bar represents the *q* value and indicates significance of the enrichment.


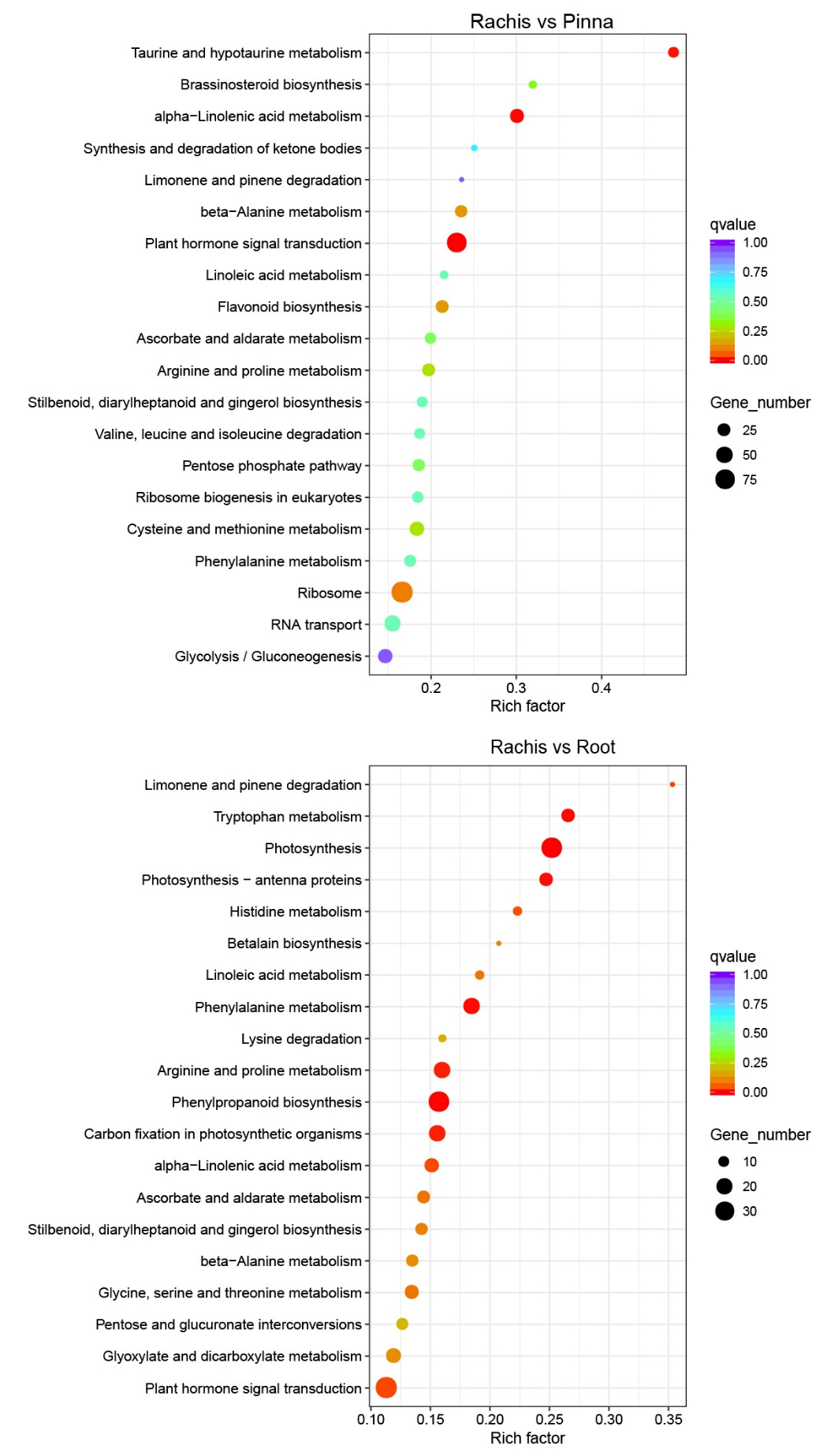


Fig. S8 Enrichment results of KEGG expression up-regulated genes in *A. latebrosa* rachis (compared with root and rachis) (top twenty). The significantly enriched pathways with corrected *p*-value (*q* value) < 0.05 were shown. Number indicates the size of the dot, describing the number of unigenes enriched in the pathway. The color bar represents the *q* value and indicates significance of the enrichment.
